# Luminescent Manganese(II) Iminophosphorane Derivatives

**DOI:** 10.3390/molecules30061319

**Published:** 2025-03-14

**Authors:** Domenico Piccolo, Jesús Castro, Daniele Rosa-Gastaldo, Marco Bortoluzzi

**Affiliations:** 1 Dipartimento di Scienze Chimiche, Università di Padova, Via Marzolo 1, 35131 Padova, Italy; domenico.piccolo@unipd.it (D.P.);; 2 Dipartimento di Scienze Molecolari e Nanosistemi, Università Ca’ Foscari Venezia, 30172 Mestre, Italy; 3 Departamento de Química Inorgánica, Facultade de Química, Universidade de Vigo, Edificio de Ciencias Experimentais, 36310 Vigo, Spain; jesusc@uvigo.gal; 4 CIRCC (Consorzio Universitario Reattività Chimica e Catalisi), Via Celso Ulpiani 27, 70126 Bari, Italy

**Keywords:** manganese, iminophosphoranes, phosphanimines, luminescence, organic-inorganic hybrids, zinc

## Abstract

The reaction between the iminophosphorane ligand *N*-phenyl-1,1,1-triphenylphosphanimine (NPh=PPh_3_) and anhydrous manganese(II) halides allowed the isolation of complexes with the general formula [MnX_2_(NPh=PPh_3_)_2_] (X = Cl, Br, I). The compounds showed luminescence in the green region attributed to the ^4^T_1_(^4^G)→^6^A_1_(^6^S) transition of the metal centre in the tetrahedral field, which was superimposed in the cases of X = Cl and X = Br on weak ligand-centred fluorescence. The emission and excitation spectra were compared with those of the free ligand and of the related zinc(II) bromo-complex. DFT calculations on the free ligand and on the manganese(II) bromo-complex helped to rationalise the experimental data. The protonation of NPh=PPh_3_ led to the formation of the iminium cation [NHPh=PPh_3_]^+^, which was used as a building block for the synthesis of organic–inorganic hybrids with the general formula [NHPh=PPh_3_]_2_[MnX_4_] (X = Cl, Br, I). The crystal structure of [NHPh=PPh_3_]_2_[MnBr_4_] was determined by means of X-ray diffraction. Green photoluminescence associated with the metal-centred transition was also observed for the organic–inorganic hybrids, with higher quantum yields with respect to the neutral [MnX_2_(NPh=PPh_3_)_2_] complexes. In the case of X = I, luminescence from the cation was superimposed on that from the tetraiodomanganate anion upon excitation of the compound with near–UV light.

## 1. Introduction

Luminescent manganese(II) derivatives are of actual interest for the development of devices based on earth-abundant elements and can have applications in several fields of advanced technology [1,2,3,4]. Organic–inorganic hybrids, where the anion is a manganese(II) halide derivative such as [MnX_4_]^2−^, [MnX_5_]^3−^, [Mn_3_X_12_]^6−^ or [MnX_3_]_n_^n−^, constitute a class of widely investigated compounds that sometimes behave as multifunctional materials. Luminescence performance strongly depends on the choice of the organic counter-cation, which is constituted in several cases by nitrogen-based species [5,6,7,8,9,10,11,12,13,14,15,16,17,18,19,20,21,22,23,24,25,26,27,28,29,30,31,32]. The photoluminescence quantum yield exceeds 95% in some cases.

Another class of extensively studied luminescent manganese(II) compounds is obtained with the introduction of suitable ligands in the coordination sphere, in particular, O-donors based on the {P=O} fragment. The tetrahedral complex [MnX_2_(O=PPh_3_)_2_], reported for the first time in 1961, represents the prototype for this family of manganese(II) derivatives [33,34,35,36,37,38]. The use of polydentate phosphine oxides allowed the preparation of compounds with improved and unusual optical features, and the studies were extended to other {P=O}-donors such as phosphoramides, arylphosphonic diamides, phosphoramidates, phosphonates and *H*-phosphinates [39,40,41,42,43,44,45,46,47,48,49,50,51,52,53,54,55,56,57,58,59,60,61,62].

The {P=NR} group of iminophosphoranes, also known as phosphanimines, is almost partly comparable to the {P=O} fragment. The phosphorus–nitrogen bond is highly polarised; thus, it is best described as a resonance hybrid between the two forms {P=NR} and {P^+^–^−^NR} [63]. The nitrogen atom can form coordinative bonds, or it can be protonated to obtain iminium cations. Examples of coordination compounds with iminophosphoranes include Groups 4–6 and 9–12 d-block elements as metal centres [64,65,66,67,68,69,70,71,72,73,74,75,76,77,78,79]. The bridging coordination mode was observed for the first time in the dinuclear carbonyl-complex [Mo_2_(CO)_6_(μ-NH=PPh_3_)_3_] [80].

Bidentate iminophosphoranes with donor moieties connected through either the nitrogen or phosphorus atoms were synthesised and applied for the preparation of d-block metal complexes [81,82,83,84,85]. It is, however, worth noting that several studies concern bis(iminophosphoranyl)methanides, i.e., the conjugate bases obtained from the deprotonation of the methylene bridge in R’N=PR_2_-CH_2_-PR_2_=NR’ species [86,87]. Examples are manganese(II) amide-complexes with the general formula [Mn{N(SiMe_3_)_2_}(ArN=PPh_2_-CH-PPh_2_=NAr)] (Ar = substituted aryl) [88,89].

The iminophoshorane moiety can also be part of multidentate species together with other donor moieties, and transition metal complexes stabilised by this type of ligands have found applications as catalysts for several organic reactions [90]. Palladium(II), platinum(II) and gold(III) derivatives of the luminescent ligand Ph_3_P=N–C_9_H_6_N (C_9_H_6_N = 8-quinolinyl) belong to this category, and they represent rare examples of iminophosphorane complexes investigated from a photophysical point of view [91].

**Scheme 1:**
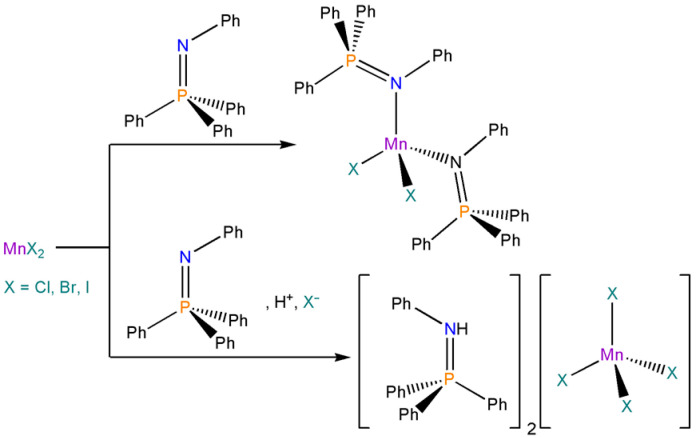
Manganese(II) iminophosphorane derivatives described in this work.

Regarding the chemistry of manganese(II) with neutral iminophosphorane ligands, the complexes [MnCl(μ-Cl)(Me_3_SiN=PEt_3_)]_2_ and [MnI_2_(Me_3_SiN=PEt_3_)_2_] were prepared through the reaction of Me_3_SiN=PEt_3_ with the proper MnX_2_ precursor and structurally characterised. The thermal treatment of [MnI_2_(Me_3_SiN=PEt_3_)_2_] led to the chelate complex [MnI_2_(Et_3_P=N-SiMe_2_-N=PEt_3_)] [92]. No study concerning the luminescence of the manganese(II) complexes was reported. Moreover, to the best of our knowledge, iminophosphorane-based manganese(II) organic–inorganic hybrids were never synthesised. In the present work, we report the synthesis and photophysical characterisation of complexes having the general formula [MnX_2_(NPh=PPh_3_)_2_] and of organic–inorganic hybrids of the type [NHPh=PPh_3_]_2_[MnX_4_] (X = Cl, Br, I), as shown in Scheme 1. The structure of [NHPh=PPh_3_]_2_[MnBr_4_] was determined by means of single-crystal X-ray diffraction.

## 2. Results and Discussion

### 2.1. Characterisation of NPh=PPh_3_ and of Its Zinc(II) Bromide Derivative

**Figure 1:**
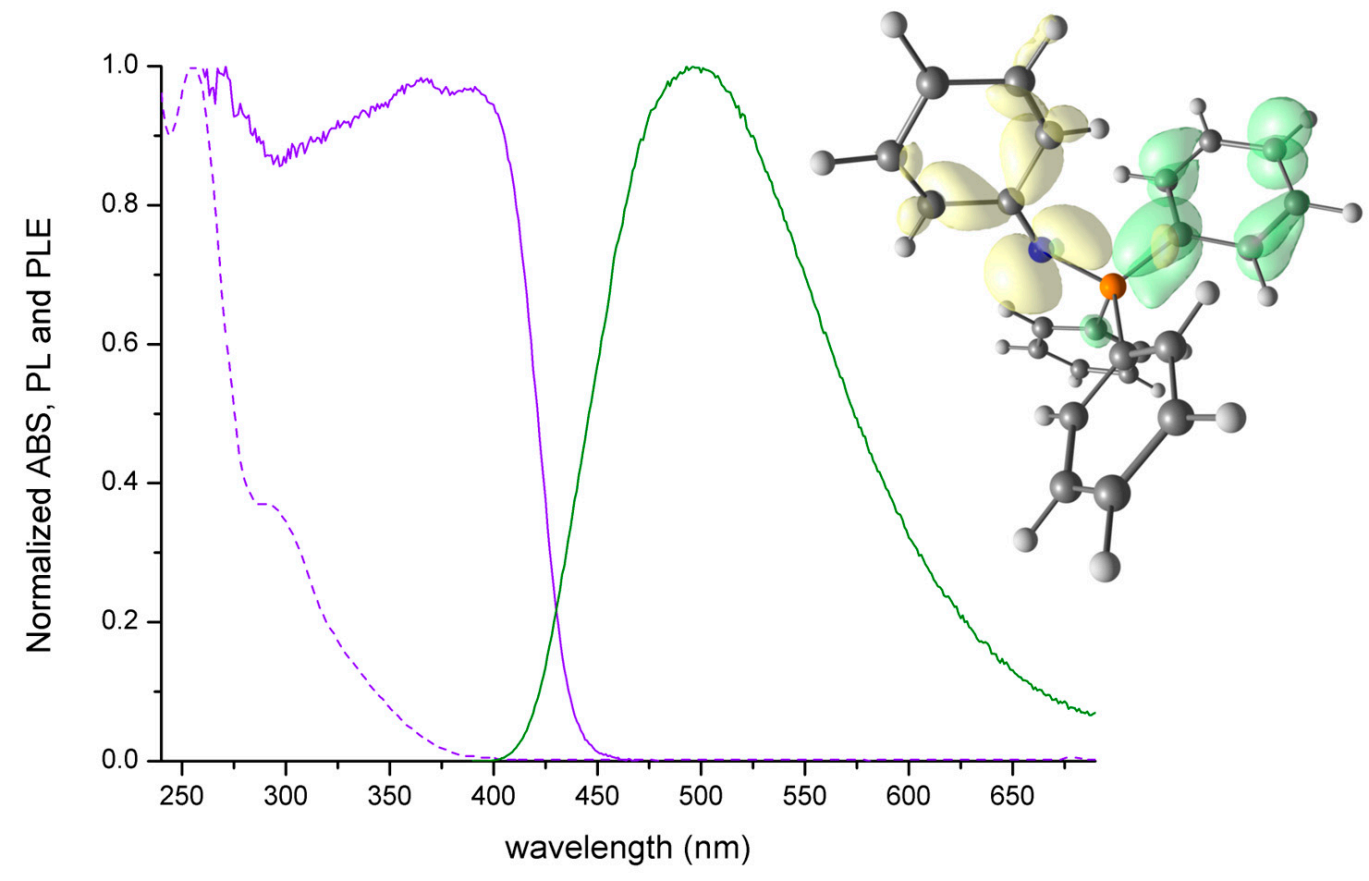
Normalised UV–VIS spectrum (violet dashed line, CH_2_Cl_2_ solution, r.t.) and normalised PL (green line, λ_ex_ = 300 nm, solid, r.t.) and PLE (violet line, λ_ex_ = 510 nm, solid, r.t.) spectra of NPh=PPh_3_. CIE 1931 chromaticity coordinates: x = 0.261, y = 0.393. First-singlet excited state geometry of NPh=PPh_3_ (P, orange; N, blue; C, grey; H, white) with hole (yellow) and electron (green) isosurfaces for the S_1_←S_0_ transition. Emission is the reverse process.

*N*-phenyl-1,1,1-triphenylphosphanimine, NPh=PPh_3_, was prepared from triphenylphosphine and phenyl azide as reported in the literature [93]. The light yellow solid showed weak photoluminescence in the green region upon UV irradiation. The compound absorbed radiation for wavelengths shorter than 400 nm, with a local maximum at about 342 nm. The excitation (PLE) range at the solid state was wider than the absorption interval in solution, since it extended up to the violet region of the visible spectrum. The emission (PL) spectrum was composed of a single, broad band centred at 496 nm (Figure 1). Time-resolved measurements allowed determination of an average lifetime value (τ) around 2 ns, which is in line with a fluorescent emission. Photoluminescence data are summarised in Table 1. The photoluminescence quantum yield (Φ) of powder samples was too low for a precise determination, and it was estimated below 1%. The weak emission did not allow determination of the Φ value of the compound in solution.

Time-dependent density functional theory (TD-DFT) calculations allowed optimisation of the first-singlet excited state of NPh=PPh_3_. The predicted value for the S_1_→S_0_ transition was 483 nm, which is in good agreement with the experimental outcomes. According to the hole–electron distribution depicted in Figure 1 [94], the transition occurred between orbitals mostly localised on the {NPh} fragment and on the P-bonded phenyl rings. The fluorescent emission thus had an intraligand charge transfer nature.

Attempts to further investigate the electronic features of NPh=PPh_3_ were carried out by means of cyclic voltammetry (Figure S1). The compound was characterised by three irreversible oxidation processes between 0.3 and 0.9 V vs. Fc^+^/Fc, with two clearly distinguishable peaks at 0.41 and 0.69 V vs. Fc^+^/Fc. The cathodic region showed an irreversible reduction peak around −2.07 V vs. Fc^+^/Fc, which was immediately followed by a dominant process attributable to electropolymerisation of the species [95].

In order to shed light on the effects of the interaction with an MX_2_ fragment on the electronic properties of NPh=PPh_3_, initial coordination chemistry studies were carried out considering anhydrous zinc(II) bromide as the metal precursor. [ZnBr_2_(NPh=PPh_3_)_2_] was prepared under mild conditions using anhydrous acetonitrile as the solvent and a 2:1 ligand/precursor ratio. All the manipulations were carried out under inert atmosphere given the limited stability of the complex in the presence of moisture. The NMR spectra of the complex showed the resonances expected for the iminophosphorane ligand, which were altered by coordination. In particular, a noticeable high-frequency shift around 29 ppm was observed for the ^31^P{^1^H} NMR resonance with respect to the free ligand (δ = 3.1 ppm, CDCl_3_, 300 K) because of the σ-donation of electron density from the {N=P} moiety to the metal centre. Another consequence of coordination is the broadening of the NMR signals, indicating fluxional behaviour in solution enhanced by the steric hindrance around zinc(II). According to literature data [86], the ν_P=N_ stretching of NPh=PPh_3_ was assigned to a strong band at 1346 cm^−1^, which was replaced by an absorption at 1240 cm^−1^ in [ZnBr_2_(NPh=PPh_3_)_2_]. Both NMR and IR data suggest that the charge-separated resonance formula Ph_3_P^+^–^−^NPh is more prevalent in the coordination compound than in the free ligand. Selected NMR and IR spectra are provided in the Supporting Materials, Figures S2 and S3.

**Figure 2:**
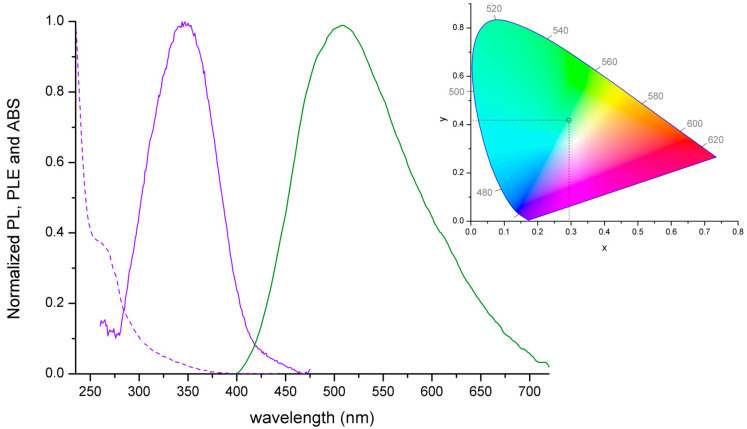
Normalised UV–VIS (violet dashed line, CH_2_Cl_2_ solution, r.t.), PL (green line, λ_ex_ = 350 nm, solid, r.t.) and PLE (violet line, λ_ex_ = 510 nm, solid, r.t.) spectra of [ZnBr_2_(NPh=PPh_3_)_2_]. CIE 1931 chromaticity diagram; chromaticity coordinates: x = 0.294, y = 0.418.

The absorption features of [ZnBr_2_(NPh=PPh_3_)_2_] did not diverge from those of the free ligand. On the other hand, the emission at the solid state was slightly red-shifted, centred around 510 nm (FWHM = 5300 cm^−1^). The luminescence fell in the green region of the CIE 1931 chromaticity diagram. The PLE spectrum showed a maximum at 347 nm, roughly corresponding to the weak lowest energy transition detected in the corresponding absorption spectrum in solution (Figure 2). Time-correlated single-photon counting (TCSPC) measurements indicated that the excited-state lifetime was in the hundreds of nanoseconds range, approximately two orders of magnitude slower than free NPh=PPh_3_. Moreover, multi-channel scaling (MSC) measurements revealed the presence of a decay in the hundreds of μs range, which was most likely related to the population of an emitting triplet state (Figure S4). It must be therefore concluded that the luminescence of [ZnBr_2_(NPh=PPh_3_)_2_] is not due to simple fluorescent decay since the experimental data support the idea of exchange between singlet and triplet excited states.

Although not common, the possible involvement of triplet states has already been documented in the recent literature for other zinc(II) derivatives [96,97,98,99]. The previously described well-separated hole and electron distributions for the free iminophosphorane suggest a scarce contribution of the exchange energy in the relative stabilisation of the triplet state, favouring the reverse intersystem crossing (RISC) process. Such a hypothesis was confirmed by calculation of the excited singlet–triplet energy gap for the free ligand, which was carried out at both the excited singlet and triplet state geometries. The values obtained were around 900 cm^−1^, and it is worth noting that the possibility of RISC is usually invoked for energy gaps below 1000 cm^−1^ [100]. Unfortunately, the non-radiative decay processes were dominant, with a photoluminescence quantum yield (Φ) around 3%. Moreover, photodecomposition was observed after prolonged irradiation with UV light.

Cyclic voltammetry measurements on [ZnBr_2_(NPh=PPh_3_)_2_] (Figure S1) showed that the oxidation processes described for NPh=PPh_3_ remained irreversible, but they were shifted at higher potential. Regarding the cathodic region, it is worth noting that the coordination to ZnBr_2_ blocks the iminophosphorane electropolymerisation, a result highlighting that the complex is quite stable in solution despite the fluxional behaviour observed by means of NMR spectroscopy.

### 2.2. Synthesis and Luminescence of [MnX_2_(NPh=PPh_3_)_2_]

Complexes having the general formula [MnX_2_(NPh=PPh_3_)_2_] (X = Cl, Br, I) were obtained in good yield by reacting NPh=PPh_3_ with the proper anhydrous MnX_2_ salt in acetonitrile at room temperature. As previously reported for the related zinc(II) complex, the stability of the compounds in the presence of water traces was limited. The characterisation data agreed with the proposed formulations. In particular, the molar magnetic susceptibility values were in line with high-spin d^5^ first-row transition metal centres and corresponded to magnetic moments between 5.8 and 6.2 BM at 293 K. The IR spectra resembled that of [ZnBr_2_(NPh=PPh_3_)_2_], with ν_P=N_ stretching assigned around 1220 cm^−1^, which shifted to lower wavenumbers with respect to the free ligand (1346 cm^−1^) because of the enhanced role of the ionic ylide resonance form. The spectra showed negligible dependence upon the choice of the coordinated halides, as shown in Figure S5. The ^31^P{^1^H} NMR spectra were composed of a single resonance in the 31–39 ppm range, which broadened because of the paramagnetic relaxation (Figure S6). The ^1^H NMR spectra show signals in the aromatic region between 8.5 and 6.5 ppm (Figure S7). The less broadened resonances are those of [MnI_2_(NPh=PPh_3_)_2_], possibly because of different correlation times (electronic relaxation or ligands exchange) when X = I [101]. The ^1^H and ^31^P NMR chemical shifts were similar to those observed for [ZnBr_2_(NPh=PPh_3_)_2_], which is in agreement with the lack of paramagnetic shift expected for Mn(II) derivatives because of the ^6^S ground state and the consequent isotropic electronic distribution [101]. The UV–VIS of [MnCl_2_(NPh=PPh_3_)_2_] and [MnBr_2_(NPh=PPh_3_)_2_] in dichloromethane were similar to that recorded for [ZnBr_2_(NPh=PPh_3_)_2_] and showed absorptions for wavelengths shorter than 350 nm, with a general blue-shift with respect to the free ligand. A shoulder around 295 nm was enhanced in the iodo-complex, and a further weak band centred at 365 was present when X = I. The spectra are shown in Figure S8 together with that of free NPh=PPh_3_. Unfortunately, we were unable to obtain single crystals suitable for X-ray diffraction.

**Figure 3:**
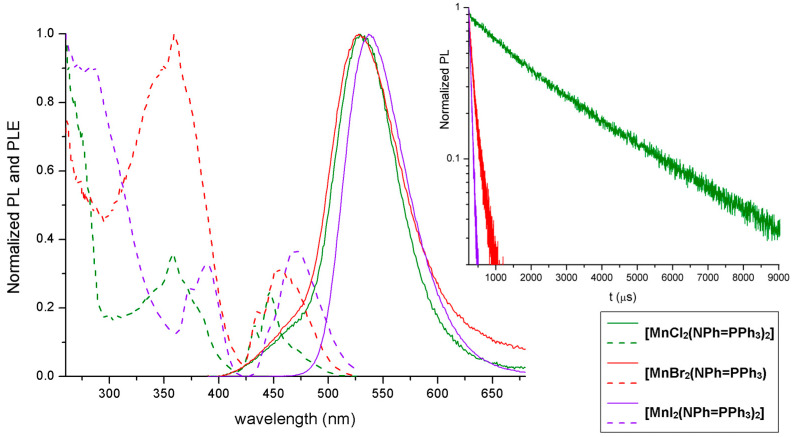
Normalised PL (solid lines, λ_ex_ = 358 nm, solid, r.t.) and PLE (dashed lines, λ_em_ = 560 nm) spectra of [MnX_2_(NPh=PPh_3_)_2_] (solid, r.t.). Luminescence decay curves (MCS, solid state, r.t., λ_ex_ = 290 nm, λ_em_ = 535 nm). CIE 1931 chromaticity coordinates: x = 0.294, y = 0.572 (X = Cl); x = 0.317, y = 0.550 (X = Br); x = 0.356, y = 0.620 (X = I). PL spectra of [MnCl_2_(NPh=PPh_3_)_2_] recorded at different excitation wavelengths are shown in Figure S9.

The formation of tetrahedral manganese(II) complexes was confirmed by the PL spectra. The three [MnX_2_(NPh=PPh_3_)_2_] compounds showed appreciable yellowish-green emission, and the related PL bands were centred between 528 and 538 nm, with FWHM values in the 2200−2900 cm^−1^ range (Figure 3). The PL bands appeared to be attributable to the ^4^T_1_(^4^G)→^6^A_1_(^6^S) transition of the metal centre in the tetrahedral field. The assignment of the metal-centred emissions was supported by the PLE spectra (Figure 3), where the transitions from the sextet ground state to the lowest-energy quartet term were present between 420 and 530 nm. The shift of the ^4^A_1_ + ^4^E(^4^G)←^6^A_1_(^6^S) transition from 432 to approximately 447 nm moving from X = Cl to X = I indicates a progressive reduction of the Racah *B* parameter. The average value, approximately 705 cm^−1^, is in line with previous data collected for other tetrahedral complexes [55]. The value reported for the free ion was 923 cm^−1^ [102]; therefore, the average nephelauxetic ratio *β* was around 0.76. The superposition of ligand- and metal-centred processes was present between 300 and 420 nm in the PLE spectra of [MnCl_2_(NPh=PPh_3_)_2_] and [MnBr_2_(NPh=PPh_3_)_2_], while the ligand-centred excitation was blue-shifted for [MnI_2_(NPh=PPh_3_)_2_]; thus, ^4^P,^4^D←^6^S transitions centred at 390 and 372 nm were clearly detectable. The luminescence decay curves shown in Figure 3 are in line with the metal-centred nature of the emissions, since the lifetime values range from tens to thousands of μs, with a clear dependence upon the choice of the halide, as commonly observed for luminescent manganese(II) halide complexes. The τ values were meaningfully reduced on increasing the halide atomic number due to acceleration of the ^4^T_1_(^4^G)→^6^A_1_(^6^S) radiative decay, which was ascribed to the increased degree of spin–orbit coupling [103].

Excitation with near–UV light of [MnCl_2_(NPh=PPh_3_)_2_] and [MnBr_2_(NPh=PPh_3_)_2_] caused the appearance of a weaker band around 460 nm. The same band was absent for the related iodo-complex. TCSPC measurements on [MnCl_2_(NPh=PPh_3_)_2_] indicated that the shoulder around 460 nm can be attributed to fluorescence decay from the coordinated ligands, with a τ value around 2 ns. The presence of free NPh=PPh_3_ was ruled out by the ^31^P{^1^H} NMR spectra. The ligand-centred emission was thus almost completely replaced by the metal-centred one, probably because of the energy transfer from the ligand-centred excited states to the Mn(II)-emitting level. The presence of only the metal-centred emission in [MnI_2_(NPh=PPh_3_)_2_] was reasonably related to the increased rate of intersystem crossing when X = I, which favoured the energy transfer.

Unfortunately, the luminescence quantum yields of the [MnX_2_(NPh=PPh_3_)_2_] were low at approximately 3% and 7% for the bromo- and iodo-complexes, respectively. The luminescence of [MnCl_2_(NPh=PPh_3_)_2_] was too low to determine the Φ value with the available experimental apparatus. Given a τ value equal to 55 μs, the radiative (k_r_) and non-radiative (k_nr_) constants for [MnI_2_(NPh=PPh_3_)_2_] were around 1.3 × 10^3^ s^−1^ and 1.7 × 10^4^ s^−1^, respectively.

The antenna effect from the coordinated iminophosphoranes on the manganese(II) centre can be proposed on the basis of the PL and PLE spectra. To give insight into this point, the structure of [MnBr_2_(NPh=PPh_3_)_2_] was simulated on the basis of DFT calculations. A tetrahedral mononuclear complex was considered for the input structure, since the metal-centred emission in the green region of the spectrum allowed exclusion of higher coordination numbers and thus the formation of [MnBr_2_(NPh=PPh_3_)_2_]_n_ polynuclear compounds of coordination polymers with bridging ligands. As shown in Figure S10, the spin density plots show that the unpaired electrons were localised on the metal centre for the sextet configuration, while a P-bonded phenyl ring was also involved in the unpaired electron density of the octet configuration. The computed energy difference between the two configurations was around 21,700 cm^−1^, while the energy of the ^4^T_1_(^4^G) level of Mn^II^ can be estimated to be around 20,400 cm^−1^ from the onset of the PL spectra, that of [MnI_2_(NPh=PPh_3_)_2_] in particular. The limited energy gap between ligand- and metal-centred excited states agrees with the possibility of ligand→metal energy transfer (ET) but also opens the possibility of the reverse process (BET). DFT calculations allowed the speculation that low quantum yields could be almost in part attributable to ligand←metal BET followed by non-radiative decay (Figure S10).

### 2.3. Synthesis of [NHPh=PPh_3_]_2_[MnX_4_], X-ray Structure Determination of the Bromo-Derivative and Luminescence Measurements

Organic–inorganic hybrids having the general formula [NHPh=PPh_3_]_2_[MnX_4_] (X = Cl, Br) were synthesised by reacting the corresponding MnX_2_ precursors with two equivalents of the iminium salts [NHPh=PPh_3_]X, which were obtained in situ from the iminophosphorane and HX in dichloromethane. [NHPh=PPh_3_]_2_[MnI_4_] was prepared by reacting a 1:2 mixture of MnI_2_ and NaI with two equivalents of [NHPh=PPh_3_][BF_4_], which were obtained in situ from NPh=PPh_3_ and HBF_4_·Et_2_O. Because of the progressive decomposition in the presence of water traces, the manipulations under air were avoided when possible. Elemental analysis data were in agreement with the proposed formulations, and the magnetic moments calculated from the molar magnetic susceptibility values at 293 K were close to the 5.9 BM expected for high-spin manganese(II) derivatives. The ^31^P{^1^H} NMR spectra were composed by a single resonance in the 36–39 ppm range (Figure S11), while the P- and N-bonded phenyl substituents were associated with broad resonances between 9.0 and 6.5 ppm in the ^1^H NMR spectra (Figure S12), without meaningful changes when varying the [MnX_4_]^2−^ anion. The ATR-IR spectra of the three compounds were almost identical, with bands in the 3170–3130 cm^−1^ range indicating the presence of N-H bonds. The spectra also showed the absence of the strong band at 1346 cm^−1^ observed for NPh=PPh_3_, which was replaced by an absorption of medium intensity around 1220 cm^−1^ (see for instance Figure S13). The spectroscopic data suggest that protonation and coordination to Mn^II^ have similar effects on the electronic structure of NPh=PPh_3_. Such an outcome was confirmed by the UV–VIS spectra since the main absorptions in solution fell at wavelengths shorter than 325 nm, which were blue-shifted compared to free NPh=PPh_3_ (Figure S14). In the case of [NHPh=PPh_3_]_2_[MnI_4_], a weak band centred at 365 nm was also present.

**Figure 4:**
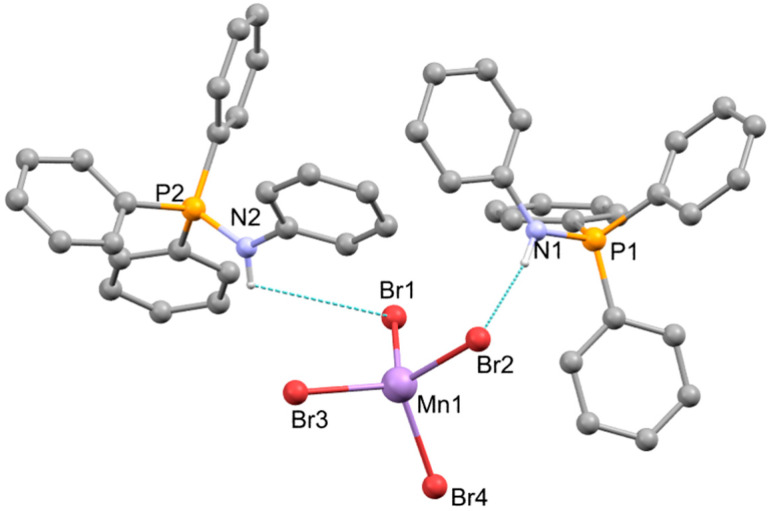
Molecular structure of [NHPh=PPh_3_]_2_[MnBr_4_]. Hydrogen atoms are not drawn, except for those on the iminium nitrogen atoms.

The formation of organic–inorganic hybrids was confirmed by the single-crystal X-ray structure determination of [NHPh=PPh_3_]_2_[MnBr_4_]. The compound crystallised in the orthorhombic space group *Pbca*. The asymmetric unit (Figure 4) contained two [NH=PPh_3_]^+^ cations and one [MnBr_4_]^2−^ dianion. Another molecule was present in the asymmetric unit, but it was not modelled, and the contribution to the reflections was eliminated by means of the usual procedure for disordered solvents molecules (see Section 3). Selected bond distances and angles are shown in Table 2.

The bond lengths around the manganese(II) centre ranged from 2.4748(11) to 2.5263(11) Å, and the angles were between 106.94(4) and 113.74(4)° and agreed well with those in similar compounds characterised by [MnBr_4_]^2−^ tetrahedral units [104]. The coordination polyhedron was studied with the SHAPE program [SHAPE V2.1] [105]. Table S1 displays the output for the geometries expected in a four vertex polyhedron [106] and indicates that the environment around manganese(II) ion is best described as an almost perfect tetrahedron. In Table S1, the classical descriptors τ_4_ and τ’_4_ for the four-coordinate geometry are also reported [107,108], and the values agree with a tetrahedral geometry around the metal. In the phosphaniminium cations, the P-N bond distances were 1.633(5) and 1.631(5) Å. These values were slightly longer than that found in the free phosphanimine, 1.603(3) Å and were similarly found in the phosphaniminium cation crystallised with [AuI_2_]^−^ anion, 1.62(2) Å, or with some borate anions such as [PhC≡CB(C_6_F_5_)_3_]^−^, 1.6357(17) Å, or [HB(C_6_F_5_)_3_]^−^, 1.6415(19) Å [109,110]. The P-N-C angles, 128.6(4) and 128.5(4)°, were between those found for the neutral phosphanimine, 130.4(3)°, and for the borate derivatives, the average value was 126.60(16)°. The angles around the phosphorus atoms ranged between 104.9(3) and 112.2(3)°, a range similar to that found in the above-mentioned phosphaniminium salts.

**Figure 5:**
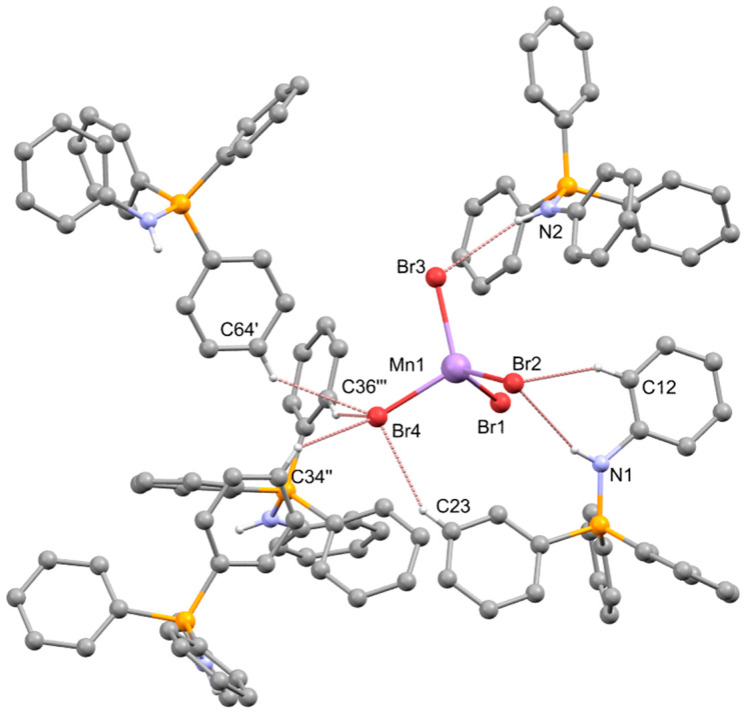
Some of the hydrogen bonds found in [NHPh=PPh_3_]_2_[MnBr_4_]. Symmetry operations: i, 3/2 − x, y + 1/2, z; ii, 1 − x, y + 1/2, 1/2 - z; iii, x + 1/2, y, 1/2 − z.

The presence of bromine atoms resulted in the formation of hydrogen bonds. The classical H-bonds involved the NH fragments, but other interactions with several aromatic CH fragments were also present. The most important are set out in Table 3, and a graphical representation is given in Figure 5. In the supramolecular network, there was also at least one π-π’-stacking interaction between two aniline rings, as shown in Figure S15, with distances between centroids of 4.136(6) Å and ring slippage of 0.898 Å. The manganese atoms in Figure S15 were separated by 11.694(2) Å, but the shortest Mn–Mn’ distance found in the crystal was 10.573(1) Å (sym. op. 3/2 − x, y + 1/2, z), as shown in Figure S16.

The powder X-ray pattern of [NHPh=PPh_3_]_2_[MnBr_4_] was comparable to that derived from the single-crystal X-ray diffraction measurement, confirming the phase purity of the compound (Figure S17).

**Figure 6:**
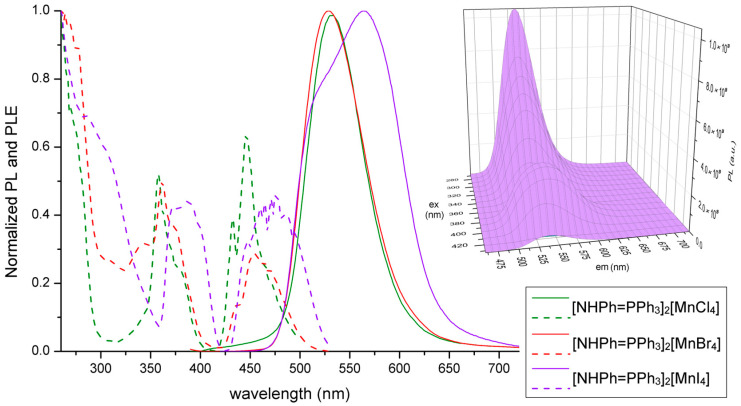
Normalised PL (solid lines, λ_ex_ = 370 nm, solid, r.t.) and PLE (dashed lines, λ_em_ = 560 nm) spectra of [NHPh=PPh_3_]_2_[MnX_4_] (solid, r.t.). Three-dimensional plot of the excitation-dependent emission of [NHPh=PPh_3_]_2_[MnI_4_] (solid, r.t.). CIE 1931 chromaticity coordinates: x = 0.309, y = 0.620 (X = Cl); x = 0.314, y = 0.631 (X = Br); x = 0.407, y = 0.558 (X = I).

**Figure 7:**
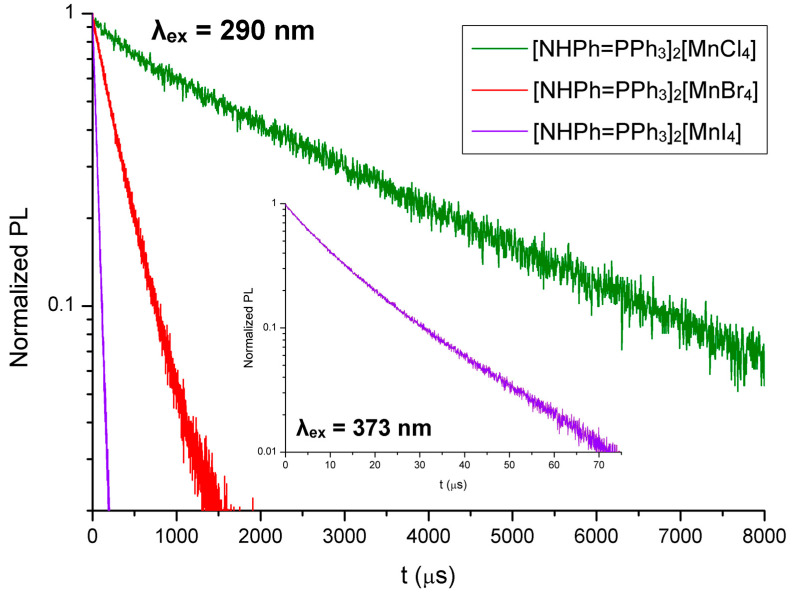
Luminescence decay curves of [NHPh=PPh_3_]_2_[MnX_4_] (MCS, λ_ex_ = 290 nm, λ_em_ = 520–535 nm; TCSPC, λ_ex_ = 373 nm, λ_em_ = 565 nm). Solid state, r.t.

The three [NHPh=PPh_3_]_2_[MnX_4_] compounds showed appreciable yellowish-green luminescence at the solid state upon excitation with UV light, particularly the bromo- and iodo-derivatives. The PL spectra of [NHPh=PPh_3_]_2_[MnCl_4_] and [NHPh=PPh_3_]_2_[MnBr_4_] were composed of a single band centred between 529 and 532 nm, with FWHM values around 2400 cm^−1^, as expected for the ^4^T_1_(^4^G)→^6^A_1_(^6^S) transition in the tetrahedral field. No dependence upon the excitation wavelength was observed, and the PLE spectra were essentially constituted by ^4^G←^6^S (420–530 nm), ^4^P,^4^D←^6^S (315–415 nm) and ^4^F←^6^S (<300 nm) transitions. The emission of [NHPh=PPh_3_]_2_[MnI_4_] samples irradiated with near–UV light was markedly different, since the metal-centred transition was superimposed on another band centred at 565 nm (Figure 6). Only the band due to the ^4^T_1_(^4^G)→^6^A_1_(^6^S) emission, centred at 537 nm, was present with excitation of the sample with radiation around 280 nm. The dependence of the PL spectrum upon the excitation wavelength is observable in the 3D plot reported in Figure 6, and the PLE spectrum revealed the superposition of the metal-centred transitions on another excitation band. Time-resolved measurements confirmed the dependence of the lifetime values from the choice of the coordinated halides, with monoexponential decays affording τ values equal to 2832 and 313 μs for the chloro- and the bromo-derivatives (Figure 7). Monoexponential decay was observed also for [NHPh=PPh_3_]_2_[MnI_4_] using λ_excitation_ equal to 290 nm, a wavelength causing only metal-centred emission. The τ value obtained from the interpolation of the decay was 49 μs. On the other hand, TCSPC measurements carried out with λ_excitation_ equal to 373 nm showed the curve reported in Figure 7. Using the previously determined τ value for the interpolation of the data, the lifetime of the second decay was estimated at around 10 μs. It is therefore likely to suppose that a phosphorescent process superimposes on the ^4^T_1_(^4^G)→^6^A_1_(^6^S) emission of the tetraiodomanganate anion when the excitation is carried out with near–UV light.

In order to shed light into the dual emission of [NHPh=PPh_3_]_2_[MnI_4_], a freshly prepared sample of [NHPh=PPh_3_][BF_4_] was synthesised from NPh=PPh_3_ and HBF_4_·Et_2_O in dichloromethane and subjected to photophysical measurements after removal of the solvent. A very weak emission centred at 515 nm (FWHM = 5900 cm^−1^) was observed upon excitation with near–UV radiation (Figure S18). The lifetime was in the nanoseconds range, which was in line with a fluorescent decay. It can be tentatively supposed that the closeness of the [MnI_4_]^2−^ anion favours the intersystem crossing in the [NHPh=PPh_3_]^+^ cations, with a red-shift of the emission of approximately 50 nm and a noticeable increase in the radiative lifetime. The same phenomenon did not occur for the other organic–inorganic hybrids because of the lower spin–orbit coupling.

As occurred for [MnCl_2_(NPh=PPh_3_)_2_], the emission of [NHPh=PPh_3_]_2_[MnCl_4_] was too low for a precise determination of the photoluminescence quantum yield. On the other hand, the Φ values of the other two organic–inorganic hybrids were between 22% and 23%, which were much higher than those of the neutral complexes. It is possible to suppose that the lack of covalent bonds between the metal centre and the iminophosphorane skeleton likely avoids the back energy transfer, thus reducing the non-radiative decay related to the vibration of the organic fragments. Given a τ value equal to 313 μs, the radiative (k_r_) and non-radiative (k_nr_) constants for [NHPh=PPh_3_]_2_[MnBr_4_] were around 1.3 × 10^3^ s^−1^ and 2.5 × 10^3^ s^−1^, respectively. k_nr_ is thus approximately one order of magnitude slower than that estimated for [MnI_2_(NPh=PPh_3_)_2_].

## 3. Experimental Section

### 3.1. Materials and Methods

Anhydrous manganese(II) and zinc(II) halides and the other inorganic and organic reactants were purchased from Merck (Darmstadt, Germany). The solvents were purified before use according to established methods [111]. Deuterated chloroform was a Euriso-Top (Saarbrücken, Germany) product, which was used as received. Phenyl azide was synthesised from phenylhydrazine under a fume hood following a reported method. The distillation step was avoided, and the compound was purified by filtration on silica gel using pentane as eluent [112]. *Safety note: organic azides are potentially explosive compounds. All the manipulations were carried out using limited quantities of reactants, with careful control of the temperature during all the synthetic steps*. *N*-phenyl-1,1,1-triphenylphosphanimine (NPh=PPh_3_) was synthesised from phenyl azide and triphenylphosphine as reported in the literature [93]. Gaseous HCl and HBr were prepared by reacting the related sodium salts with concentrated sulfuric acid and they were absorbed in dichloromethane. The concentration was determined by titration immediately before use.

Moisture-sensitive compounds such as NPh=PPh_3_, its salts and coordination compounds were prepared and stored in a MBraun (Garching bei München, Germany) MB10 glove box filled with nitrogen and equipped for organic and inorganic syntheses.

### 3.2. Characterisations

Carbon, hydrogen and nitrogen elemental analyses were performed using an Elementar (Langenselbold, Germany) Unicube microanalyzer. The halide contents were determined by means of Mohr’s method [113]. Magnetic susceptibility measurements were carried out on solid samples at 298 K and 3.5 kGauss magnetic field strength using an MK1 magnetic susceptibility balance (Sherwood Scientific Ltd., Cambridge, UK). The measured magnetic susceptibility was corrected for the diamagnetic contribution using tabulated Pascal’s constants [114]. IR spectra of the neutral species dispersed in KBr (spectroscopy grade, Merck) were collected in the 4000–450 cm^−1^ range using a Perkin–Elmer (Shelton, CT, USA) Spectrum One spectrophotometer. The samples were prepared in a glove box. ATR-IR spectra of the ionic compounds were recorded with a Perkin–Elmer Spectrum Two spectrophotometer equipped with diamond ATR. ^1^H and ^31^P{^1^H} NMR spectra were collected employing a Bruker Avance 400 instrument (Billerica, MA, USA) operating at 400.13 MHz of ^1^H resonance. ^1^H NMR spectra were referenced to the partially non-deuterated fraction of the solvent, which is itself quoted to tetramethylsilane. ^31^P chemical shifts are referred to external 85% H_3_PO_4_ in water. Cyclic voltammetry measurements in acetone containing 0.1 M LiClO_4_ were conducted using an eDAQ (Denistone, Australia) ET014-199 instrument coupled with an eDAQ ET074-1 glassy carbon working electrode (1 mm diameter) and an eDAQ ET078-1 Pt-coated titanium rod auxiliary electrode. All the measurements were carried out under argon at room temperature. Ferrocene was introduced as an internal standard, and a Pt wire was used as pseudo-reference electrode. CV data are reported following the IUPAC convention [115].

### 3.3. Synthesis of [ZnBr_2_(NPh=PPh_3_)_2_]

Anhydrous ZnBr_2_ (0.113 g, 0.5 mmol) was dissolved in 25 mL of acetonitrile, and then NPh=PPh_3_ (0.353 g, 1.0 mmol) was added under stirring. The reaction mixture was kept under stirring at room temperature for 12 h, and then the solvent was removed by evaporation at reduced pressure. Dichloromethane (25 mL) was added, and the solution was purified by centrifugation. After evaporation under reduced pressure, diethyl ether (10 mL) was added, and the solid that separated was collected by filtration, washed with 5 mL of diethyl ether and dried under a vacuum. The yield was 0.303 g (65%). 

*Characterisation of [ZnBr_2_(NPh=PPh_3_)_2_]*. Anal. calcd. for C_48_H_40_Br_2_N_2_P_2_Zn (931.99 g mol^−1^, %): C, 61.86; H, 4.33; N, 3.01; Br, 17.15. Found (%): 61.62; H, 4.35; N, 2.98; Br, 17.08. IR (KBr, cm^−1^): 1249 sh, 1240 m (ν_PN_). ^1^H NMR (CDCl_3_, 300 K) δ 8.00–7.35 (m, br, 15H, P-Ph), 7.15–6.80 (m, br, 5H, N-Ph). ^31^P{^1^H} NMR (CDCl_3_, 300 K) δ 32.0 (s, FWHM = 100 Hz).

### 3.4. Synthesis of [MnX_2_(NPh=PPh_3_)_2_] (X = Cl, Br, I)

[MnX_2_(NPh=PPh_3_)_2_] complexes (X = Cl, Br, I) were synthesised as described for [ZnBr_2_(NPh=PPh_3_)_2_] using 0.5 mmol of the proper manganese(II) halide (X = Cl, 0.063 g; X = Br, 0.107 g; X = I, 0.154 g). The solubilisation of MnCl_2_ before the addition of the ligand was favoured by heating to reflux the reaction mixture for 30 min. Yields: X = Cl, 0.246 g (59%); X = Br, 0.327 g (71%); X = I, 0.355 g (70%).

*Characterisation of [MnCl_2_(NPh=PPh_3_)_2_]*. Anal. calcd. for C_48_H_40_Cl_2_N_2_P_2_Mn (832.64 g mol^−1^, %): C, 69.24; H, 4.84; N, 3.36; Cl, 8.52. Found (%): 68.95; H, 4.87; N, 3.30; Cl, 8.48. χ_M_^corr^ (cgsu): 1.65 × 10^−2^. μ (293 K, BM): 6.2. IR (KBr, cm^−1^): 1220 m (ν_PN_). ^31^P{^1^H} NMR (CDCl_3_, 300 K) δ 38.0 (s, FWHM = 370 Hz).

*Characterisation of [MnBr_2_(NPh=PPh_3_)_2_]*. Anal. calcd. for C_48_H_40_Br_2_N_2_P_2_Mn (921.54 g mol^−1^, %): C, 62.56; H, 4.38; N, 3.04; Br, 17.34. Found (%): 62.32; H, 4.41; N, 3.00; Br, 17.27. χ_M_^corr^ (cgsu): 1.60 × 10^−2^. μ (293 K, BM): 6.1. IR (KBr, cm^−1^): 1226 m, 1222 m (ν_PN_). ^31^P{^1^H} NMR (CDCl_3_, 300 K) δ 36.2 (s, FWHM = 120 Hz). 

*Characterisation of [MnI_2_(NPh=PPh_3_)_2_]*. Anal. calcd. for C_48_H_40_I_2_N_2_P_2_Mn (1015.54 g mol^−1^, %): C, 56.77; H, 3.97; N, 2.76; I, 24.99. Found (%): 56.55; H, 4.00; N, 2.73; I, 25.05. χ_M_^corr^ (cgsu): 1.46 × 10^−2^. μ (293 K, BM): 5.8. IR (KBr, cm^−1^): 1217 m (ν_PN_). ^31^P{^1^H} NMR (CDCl_3_, 300 K) δ 31.8 (s, FWHM = 150 Hz).

### 3.5. Synthesis of [NHPh=PPh_3_)]_2_[MnX_4_] (X = Cl, Br)

A freshly prepared 2.0 M solution of HX (X = Cl, Br) in dichloromethane (0.5 mL) was added to 0.353 g (1.0 mmol) of NPh=PPh_3_ dissolved in 10 mL of dichloromethane. After 30 min under stirring at room temperature, the solution was added to an acetonitrile solution (20 mL) containing 0.5 mmol of anhydrous MnX_2_ (X = Cl, 0.063 g; X = Br, 0.107 g). The resulting reaction mixture was left under stirring for 12 h, then the solvents were removed by evaporation under reduced pressure and diethyl ether (10 mL) was added. The solid products were collected by filtration, washed with 5 mL of diethyl ether and dried under vacuum. Yields: X = Cl, 0.403 g (89%); X = Br, 0.493 g (91%). Crystals of [NHPh=PPh_3_)]_2_[MnBr_4_] suitable for X-ray diffraction were obtained by slow diffusion of diethyl ether in dichloromethane solutions.

*Characterisation of [NHPh=PPh_3_)]_2_[MnCl_4_]*. Anal. calcd. for C_48_H_42_Cl_4_MnN_2_P_2_ (905.56 g mol^−1^, %): C, 63.66; H, 4.67; N, 3.09; Cl, 15.66. Found (%): 63.40; H, 4.70; N, 3.05; Cl, 15.72. χ_M_^corr^ (cgsu): 1.45 × 10^−2^. μ (293 K, BM): 5.8. ATR-IR (cm^−1^): 3134 (ν_NH_), 1220 (ν_PN_). ^31^P{^1^H} NMR (CDCl_3_, 300 K) δ 38.8 (FWHM = 190 Hz).

*Characterisation of [NHPh=PPh_3_)]_2_[MnBr_4_]*. Anal. calcd. for C_48_H_42_Br_4_MnN_2_P_2_ (1083.36 g mol^−1^, %): C, 53.22; H, 3.91; N, 2.59; Br, 29.50. Found (%): 53.11; H, 3.88; N, 2.55; Br, 29.61. χ_M_^corr^ (cgsu): 1.48 × 10^−2^. μ (293 K, BM): 5.9. ATR-IR (cm^−1^): 3134 (ν_NH_), 1219 (ν_PN_). ^31^P{^1^H} NMR (CDCl_3_, 300 K) δ 37.8 (FWHM = 260 Hz).

### 3.6. Synthesis of [NHPh=PPh_3_)]_2_[MnI_4_]

Anhydrous MnI_2_ (0.309 g, 1.0 mL) was dissolved in 30 mL of acetonitrile together with 0.300 g (2.0 mmol) of NaI. In another flask, NPh=PPh_3_ (0.707 g, 2.0 mmol) was dissolved in 20 mL of dichloromethane, and HBF_4_·Et_2_O (275 μL, 2.0 mmol) was added. After 30 min under stirring at room temperature, the two solutions were mixed and left under stirring at room temperature for 12 h. The reaction mixture was purified by centrifugation, and then the solvents were evaporated under reduced pressure. The addition of diethyl ether (10 mL) caused the separation of a solid that was filtered, washed with 5 mL of diethyl ether and dried under a vacuum. The yield was 0.439 g (69%).

*Characterisation of [NHPh=PPh_3_)]_2_[MnI_4_]*. Anal. calcd. for C_48_H_42_I_4_MnN_2_P_2_ (1271.36 g mol^−1^, %): C, 45.35; H, 3.33; N, 2.20; I, 39.93. Found (%): 45.15; H, 3.29; N, 2.15; I, 40.08. χ_M_^corr^ (cgsu): 1.42 × 10^−2^. μ (293 K, BM): 5.8. ATR-IR (cm^−1^): 3169 (ν_NH_), 1225 (ν_PN_). ^31^P{^1^H} NMR (CDCl_3_, 300 K) δ 36.4 (FWHM = 80 Hz).

### 3.7. Absorption and Luminescence Measurements

Absorption spectra in dichloromethane at room temperature were recorded in a 10 mm × 10 mm quartz cuvette (Hellma GmbH, Müllheim, Germany) with a Yoke 6000Plus double-beam spectrophotometer (Fengxian, China). Photoluminescence emission (PL) and excitation (PLE) spectra as well as lifetime decay curves were registered on solid samples at room temperature using a Horiba Jobin Yvon (Kyoto, Japan) Fluorolog-3 spectrofluorometer. Air-tight quartz sample holders were used and filled in the glove box to avoid interactions of the air-sensitive complexes with moisture. A continuous wave xenon arc lamp was used as the source, and the excitation wavelength was selected using a double Czerny–Turner monochromator. Suitable long-pass filters were placed in front of the acquisition systems. The detector was composed of a single Horiba (Kyoto, Japan) iHR 320 monochromator and a Hamamatsu (Shizuoka, Japan) R928 photomultiplier tube. The excitation and emission spectra were corrected for the instrumental functions. Time-resolved analyses were performed in multi-channel scaling mode (MCS) or time-correlated single photon counting mode (TCSPC) employing Horiba SpectraLED and NanoLED pulsed sources. The room-temperature photoluminescence quantum yields (Φ) at the solid state were measured employing an OceanOptics (Orlando, FL, USA) HR4000CG UV–vis–NIR detector fibre-coupled to an integrating sphere connected to OceanOptics UV LED continuous sources (λ_max_ = 310 and 365 nm). The values are reported as the average of three measurements.

### 3.8. Single Crystal X-ray Structure Determination and Powder X-ray Measurements

Crystallographic data relative to [NHPh=PPh_3_]_2_[MnBr_4_] were collected at CACTI (Universidade de Vigo) with a Bruker (Billerica, MA, USA) D8 Venture Photon II CMOS detector. The Mo-Kα radiation (λ = 0.71073 Å) was generated by an Incoatec (Geesthacht, Germany) Microfocus Source IµS. The software APEX version 2022.1-1 was used for collecting frames of data, indexing reflections and determination of lattice parameters, SAINT version 8.40B was used for integration of the intensity of reflections, and SADABS version 2016/2 was used for scaling and empirical absorption correction [116]. The crystallographic treatment was performed with the Oscail version 4.7.1 program [117] and solved using the SHELXT version 2018/2 program [118]. The structure was subsequently refined by a full-matrix least-squares based on *F*^2^ using the SHELXL version 2019/2 program [119]. Non-hydrogen atoms were refined with anisotropic displacement parameters. A disorder solvent was found in the final density map, but its contribution to the reflections was eliminated by using the usual SQUEEZE procedure of PLATON version 110423 [120]. The same PLATON suite was also used to obtain some geometrical parameters from the cif file. Further details concerning crystal data and structural refinement are given in Table 4. CCDC 2421580 contains the supplementary crystallographic data for this paper. These data can be obtained free of charge from the Cambridge Crystallographic Data Centre via https://www.ccdc.cam.ac.uk/structures/ (accessed on 10 March 2025). Powder X-ray measurements were carried out at room temperature with a Malvern Panalytical (Malvern, UK) Empirean diffractometer.

### 3.9. Computational Details

Computational geometry optimisations were carried out using the PBEh-3c method, which is a reparametrised version of the hybrid-GGA PBE0 functional (with 42% HF exchange) that uses a split-valence double-zeta basis set (def2-mSVP) and adds three corrections considering dispersion, basis set superposition and other basis set incompleteness effects [121,122,123,124]. The stationary points were characterised as local minima my means of IR simulations (harmonic approximation). The same method was applied for TD-DFT calculations, which were carried out considering 16 states. The unrestricted approach was used for compounds containing unpaired electrons, and the absence of meaningful spin contamination was verified by comparing the computed <*S*^2^> values with the theoretical ones. The software used was ORCA version 5.0.3 [125]. The output was elaborated using MultiWFN, version 3.8 [94,126,127]. Cartesian coordinates of the DFT-optimised structures are provided in the Supporting Materials, Lists S1–S4.

## 4. Conclusions

Manganese(II) iminophosphorane derivatives were subjected to an investigation of their photophysical features for the first time. The ligand *N*-phenyl-1,1,1-triphenylphosphanimine was revealed to be suitable for the preparation of luminescent tetrahedral manganese(II) halide complexes, with sensitisation of the metal-centred luminescence. The photoluminescence quantum yields of the neutral complexes resulted, however limited, most likely because of metal-to-ligand back energy transfer followed by non-radiative decay. The protonation of *N*-phenyl-1,1,1-triphenylphosphanimine led to the formation of the corresponding iminium cation, from which luminescent organic–inorganic hybrids containing tetrahedral tetrahalomanganate anions were prepared. The photoluminescence quantum yield values were higher than those of the corresponding neutral complexes but were unfortunately lower than the best results reported in the recent literature. The present work, however, highlighted the versatility of iminophosphoranes for the preparation of manganese(II) luminescent derivatives, and future research will be focused on the replacement of the N- and P-bonded substituents in the NR’=PR_3_ skeleton to improve the luminescent features. Bidentate iminophosphoranes will be also subjected to investigation in this field.

## Figures and Tables

**Table 1:** Room-temperature photophysical data. UV–VIS data for CH_2_Cl_2_ solutions; PL, PLE, τ and Φ data for solid samples.

Compound	Photoluminescence Data
**NPh=PPh_3_**	**UV-VIS** (nm): <400, 342 sh, 298 sh, 256. **PL** (λ_ex_ = 300 nm, nm): 496 (FWHM = 4800 cm^−1^). **PLE** (λ_em_ = 510 nm, nm): <450. **τ** (λ_ex_ = 373 nm, λ_em_ = 415 nm, ns, TCSPC): 2.
**[ZnBr_2_(NPh=PPh_3_)_2_**]	**UV-VIS** (nm): <400, 335 sh, 262 sh. **PL** (λ_ex_ = 350 nm, nm): 510 (FWHM = 5300 cm^−1^). **PLE** (λ_em_ = 510 nm, nm): <470, max 347. **τ** (λ_ex_ = 373 nm, λ_em_ = 510 nm, ns, TCSPC): 222 (av.). **τ** (λ_ex_ = 290 nm, λ_em_ = 510 nm, μs, MCS): 303 (av.). **Φ**: 3%.
**[MnCl_2_(NPh=PPh_3_)_2_]**	**UV-VIS** (nm): <350, 295 sh, 276 sh, 269, 264. **PL** (λ_ex_ = 358 nm, nm): 460 sh, 530 (FWHM = 2400 cm^−1^). **PLE** (λ_em_ = 560 nm, nm): 470, 446, 432, 420–300 (358 max), <295. **τ** (λ_ex_ = 373 nm, λ_em_ = 410 nm, ns, TCPSC): 2. **τ** (λ_ex_ = 290 nm, λ_em_ = 535 nm, μs, MCS): 2478.
**[MnBr_2_(NPh=PPh_3_)_2_]**	**UV-VIS** (nm): <350, 295 sh, 276 sh, 268, 261. **PL** (λ_ex_ = 358 nm, nm): 528 (FWHM = 2700 cm^−1^). **PLE** (λ_em_ = 560 nm, nm): 470 sh, 454, 435, 420–300 (359 max), <295. **τ** (λ_ex_ = 373 nm, λ_em_ = 410 nm, ns, TCSPC): 2. **τ** (λ_ex_ = 290 nm, λ_em_ = 535 nm, μs, MCS): 151. **Φ**: 3%.
**[MnI_2_(NPh=PPh_3_)_2_]**	**UV-VIS** (nm): <400, 364, 292, 276, 268. **PL** (λ_ex_ = 358 nm, nm): 538 (FWHM = 2200 cm^−1^). **PLE** (λ_em_ = 560 nm, nm): 472, 447 sh, 390, 372, <360. **τ** (λ_ex_ = 290 nm, λ_em_ = 535 nm, μs, MCS): 55. **Φ**: 7%.
**[NHPh=PPh_3_]_2_[MnCl_4_]**	**UV-VIS** (nm): 299 sh, 276, 269, 262. **PL** (λ_ex_ = 370 nm, nm): 532 (FWHM = 2300 cm^−1^). **PLE** (λ_em_ = 560 nm, nm): 467 sh, 448, 432, 382 sh, 371 sh, 358, <295. **τ** (λ_ex_ = 290 nm, λ_em_ = 530 nm, μs, MCS): 2832.
**[NHPh=PPh_3_]_2_[MnBr_4_]**	**UV-VIS** (nm): 299 sh, 276, 269, 262. **PL** (λ_ex_ = 370 nm, nm): 529 (FWHM = 2500 cm^−1^). **PLE** (λ_em_ = 560 nm, nm): 471 sh, 454, 436, 375 sh, 362, 343, 312, <295. **τ** (λ_ex_ = 290 nm, λ_em_ = 535 nm, μs, MCS): 313. **Φ**: 22%.
**[NHPh=PPh_3_]_2_[MnI_4_]**	**UV-VIS** (nm): 365, 299 sh, 276, 269, 262, <255. **PL** (λ_ex_ = 370 nm, nm): 565, 525 sh. **PL** (λ_ex_ = 280 nm, nm): 537 (FWHM = 2200 cm^−1^). **PLE** (λ_em_ = 560 nm, nm): 471, 446 sh, 390, 373, <355. **τ** (λ_ex_ = 290 nm, λ_em_ = 520 nm, μs, MCS): 49. **τ** (λ_ex_ = 373 nm, λ_em_ = 565 nm, μs, MCS): 10 (55%), 49 ^a^ (45%). **Φ**: 23%.

^a^ Fixed value in the biexponential fit.

**Table 2:** Selected bond lengths [Å] and angles [°] for [NHPh=PPh_3_]_2_[MnBr_4_].

Mn(1)-Br(1)	2.5055(10)	Mn(1)-Br(3)	2.4916(11)
Mn(1)-Br(2)	2.5263(11)	Mn(1)-Br(4)	2.4748(11)
P(1)-N(1)	1.633(5)	P(2)-N(2)	1.631(5)
P(1)-C(31)	1.785(5)	P(2)-C(61)	1.773(6)
P(1)-C(21)	1.786(6)	P(2)-C(71)	1.783(7)
P(1)-C(41)	1.795(6)	P(2)-C(81)	1.798(7)
N(1)-C(11)	1.423(7)	N(2)-C(51)	1.421(8)
Br(1)-Mn(1)-Br(2)	106.94(4)	Br(3)-Mn(1)-Br(1)	109.01(4)
Br(4)-Mn(1)-Br(1)	108.17(4)	Br(3)-Mn(1)-Br(2)	113.74(4)
Br(4)-Mn(1)-Br(2)	108.77(4)	Br(4)-Mn(1)-Br(3)	110.03(4)
N(1)-P(1)-C(31)	111.2(2)	N(2)-P(2)-C(61)	104.9(3)
N(1)-P(1)-C(21)	106.5(2)	N(2)-P(2)-C(71)	112.2(3)
N(1)-P(1)-C(41)	111.8(3)	N(2)-P(2)-C(81)	111.6(3)
C(11)-N(1)-P(1)	128.6(4)	C(51)-N(2)-P(2)	128.5(4)

**Table 3:** Hydrogen bonds for [NHPh=PPh_3_]_2_[MnBr_4_] [Å and °].

D-H…A	(D-H)	d(H…A)	d(D…A)	<(DHA)
N(1)-H(1)…Br(2)	0.86	2.76	3.586(5)	161.9
C(12)-H(12)…Br(2)	0.93	2.96	3.757(7)	144.5
N(2)-H(2)…Br(3)	0.86	2.68	3.520(5)	164.8
C(23)-H(23)…Br(4)	0.93	2.89	3.720(7)	149.6
C(64i)-H(64i)…Br(4)	0.93	2.97	3.658(8)	132.3
C(34ii)-H(34ii)…Br(4)	0.93	2.96	3.627(7)	130.0
C(36iii)-H(36iii)…Br(4)	0.93	2.91	3.769(6)	154.1

Symmetry operations used: i, 3/2 − x, y + 1/2, z; ii, 1 − x, y + 1/2, 1/2 − z; iii, x + 1/2, y, 1/2 − z.

**Table 4:** Crystal data and structure refinement for [NHPh=PPh_3_]_2_[MnBr_4_].

Empirical formula	C_48_ H_42_ Br_4_ Mn N_2_ P_2_	Index ranges	−18 ≤ *h* ≤ 21
Moiety formula	(C_24_ H_21_ N P)_2_(Mn Br_4_)		−18 ≤ *k* ≤ 18
Formula weight	1083.35		−47 ≤ *l* ≤ 44
Temperature	296(2) K	Reflections collected	95,854
Wavelength	0.71073 Å	Independent reflections	9780 [*R*_int_ = 0.0581 *R*σ = 0.0382]
Crystal system	Orthorhombic	Reflections observed (>2σ)	6622
Space group	*Pbca*	Data completeness	0.999
Unit cell dimensions	a = 17.7894(8) Å	Absorption correction	Semi-empirical from equivalents
	b = 15.3223(6) Å	Max. and min. transmission	0.8620 and 0.6668
	c = 39.1289(17) Å	Refinement method	Full-matrix least-squares on *F*^2^
Volume	10,665.5(8) Å3	Data/restraints/parameters	9780/0/514
Z	8	Goodness-of-fit on *F*^2^	1.048
Density (calculated)	1.349 Mg/m^3^	Final R indices [*I* > 2σ(*I*)]	*R*_1_ = 0.0548
Absorption coefficient	3.335 mm^−1^		w*R*_2_ = 0.1363
F(000)	4312	R indices (all data)	*R*_1_ = 0.0862
Crystal size	0.141 mm × 0.092 mm × 0.048 mm		w*R*_2_ = 0.1508
Theta range for data collection	2.082 to 25.368°	Largest diff. peak and hole	0.516 and −0.521 e.Å^−3^

## Data Availability

Data are contained within the article and Supplementary Materials.

## Supplementary Materials

The following supporting information can be downloaded at https://www.mdpi.com/article/10.3390/molecules30061319/s1: Figure S1: Cyclic voltammograms of [ZnBr_2_(NPh=PPh_3_)_2_] and NPh=PPh_3_; Figure S2: ^1^H NMR and ^31^P{^1^H} NMR spectra of [ZnBr_2_(NPh=PPh_3_)_2_]; Figure S3: IR spectra of [ZnBr_2_(NPh=PPh_3_)_2_] and NPh=PPh_3_; Figure S4: Luminescence decay curves of [ZnBr_2_(NPh=PPh_3_)_2_]; Figure S5: IR spectra (KBr) of [MnX_2_(NPh=PPh_3_)_2_] (X = Cl, Br, I) and of NPh=PPh_3_; Figure S6: ^31^P{^1^H} NMR spectra of [MnX_2_(NPh=PPh_3_)_2_]; Figure S7: ^1^H NMR spectra of [MnX_2_(NPh=PPh_3_)_2_]; Figure S8: UV-VIS spectra of [MnX_2_(NPh=PPh_3_)_2_] (X = Cl, Br, I) and of NPh=PPh_3_; Figure S9: PL spectra of [MnCl_2_(NPh=PPh_3_)_2_] recorded at different excitation wavelengths; Figure S10: DFT-optimised structures of [MnBr_2_(NPh=PPh_3_)_2_], sextet and octet configurations, with spin density surfaces and energy differences between the two structures compared to the ^4^T_1_(^4^G)–^6^A_1_(^6^S) experimental energy gap; Figure S11: ^31^P{^1^H} NMR spectra of [NHPh=PPh_3_]_2_[MnX_4_]; Figure S12: ^1^H NMR spectra of [NHPh=PPh_3_]_2_[MnX_4_]; Figure S13 ATR-IR spectrum of [NHPh=PPh_3_]_2_[MnI_4_]; Figure S14: UV–VIS spectra of [NHPh=PPh_3_]_2_[MnX_4_] (X = Cl, Br, I) and of NPh=PPh_3_; Table S1: Output of the SHAPE software and four-coordinate geometry indexes τ_4_ and τ’_4_; Figure S15: π,π’-stacking interaction; Figure S16: Unit cell content showing the shortest distance between two Mn atoms; Figure S17: Comparison of the X-ray patterns of [NHPh=PPh_3_]_2_[MnBr_4_] obtained from powder and single crystal diffraction measurements; Figure S18: Normalised PL and PLE spectra of [NHPh=PPh_3_][BF_4_]; List S1: Cartesian coordinates of NPh=PPh_3_, ground state; List S2: Cartesian coordinates of NPh=PPh_3_, first singlet excited state; List S3: Cartesian coordinates of [MnBr_2_(NPh=PPh_3_)_2_], sextet state; List S4: Cartesian coordinates of [MnBr_2_(NPh=PPh_3_)_2_], octet state.
