# Supplementary material for: Luminescent Manganese(II) Iminophosphorane Derivatives
Source: Molecules. 2025 Mar 14;30(6):1319. doi: 10.3390/molecules30061319 (PMC11945198; doi:10.3390/molecules30061319)
Supplement: Supplementary file 1 [file molecules-30-01319-s001.zip › molecules-3505558-supplementary.pdf]

Luminescent Manganese(II) Iminophosphorane Derivatives

Domenico Piccolo [1,2], Jesús Castro [3], Daniele Rosa-Gastaldo [1] and Marco Bortoluzzi [2,4,*]

Supplementary Materials

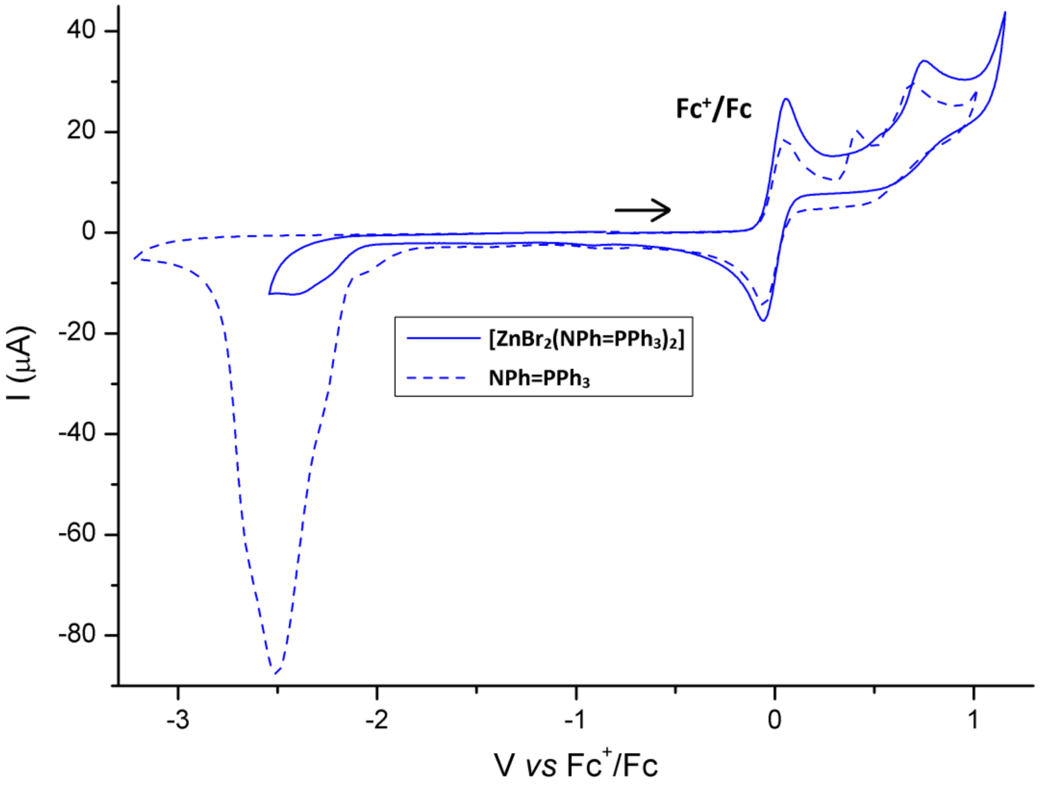

Figure S1. Cyclic voltammograms of $[ZnBr_2(NPh{=}PPh_3)_2]$ and $NPh{=}PPh_3$. Acetone/$LiClO_4$, r.t., Ar atmosphere, glassy carbon electrode, ferrocene (Fc) as internal reference, scan rate 250 mV/s.

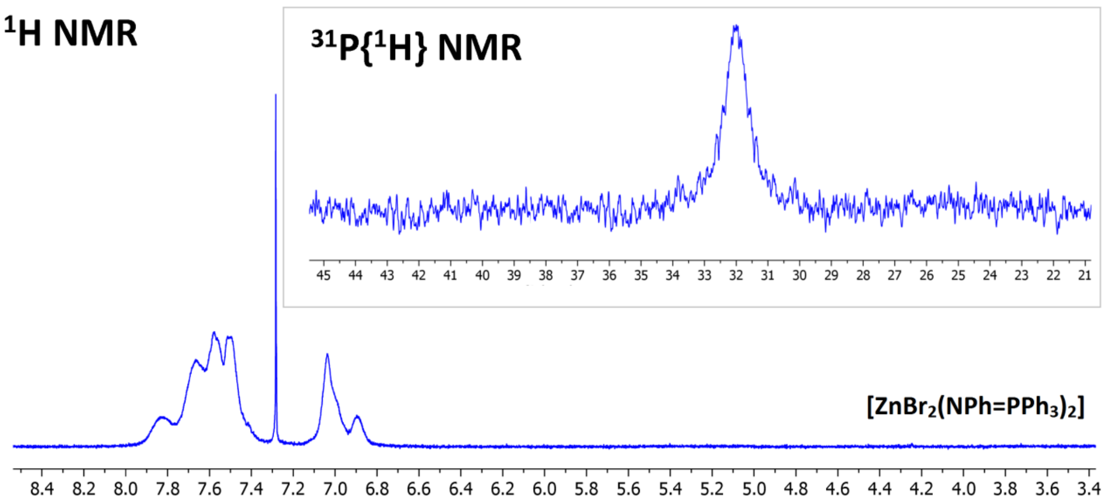

Figure S2. ^1H NMR and $^{31}P\{^1H\}$ NMR spectra of $[ZnBr_2(NPh{=}PPh_3)_2]$ ($CDCl_3$, 300 K).

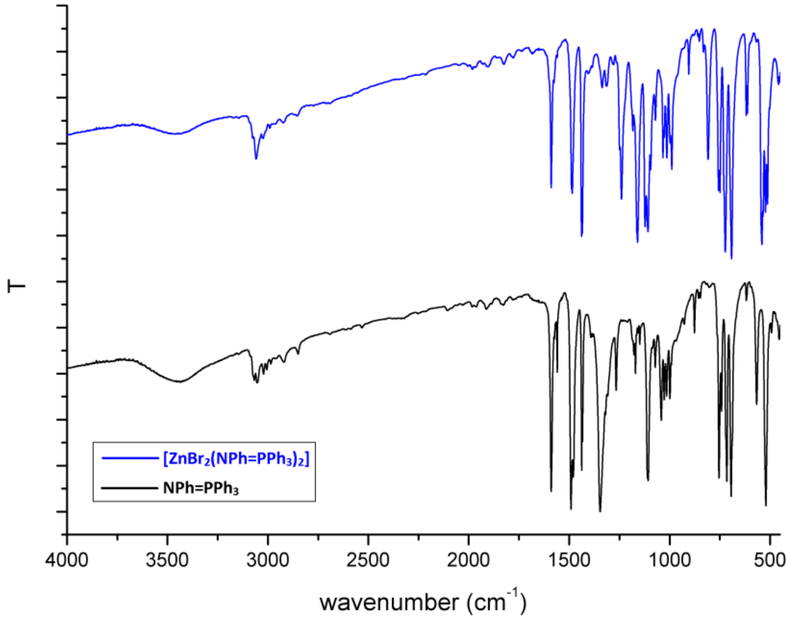

Figure S3. IR spectra (KBr) of $[ZnBr_2(NPh{=}PPh_3)_2]$ and $NPh{=}PPh_3$.

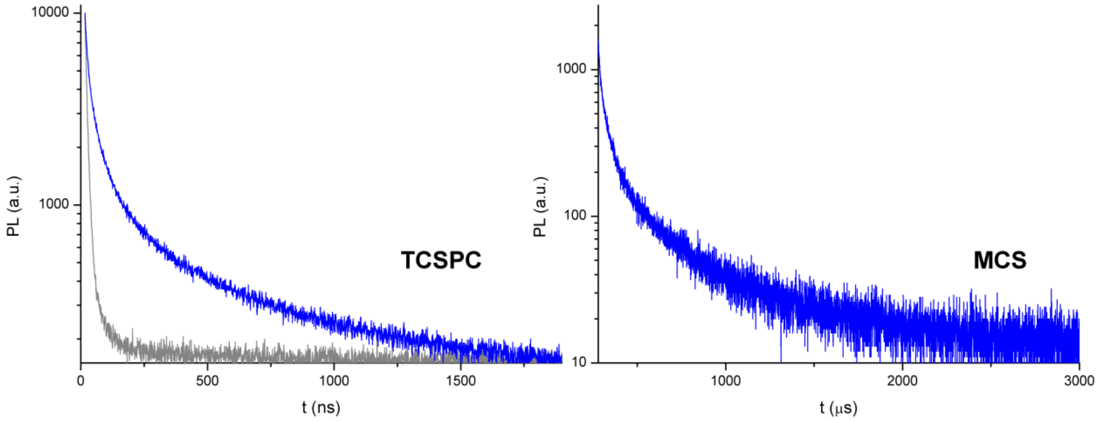

Figure S4. Luminescence decay curves of $[ZnBr_2(NPh{=}PPh_3)_2]$. Solid state, r.t., λ_{ex} = 373 nm (TCSPC), 290 nm (MCS), λ_{em} = 510 nm.

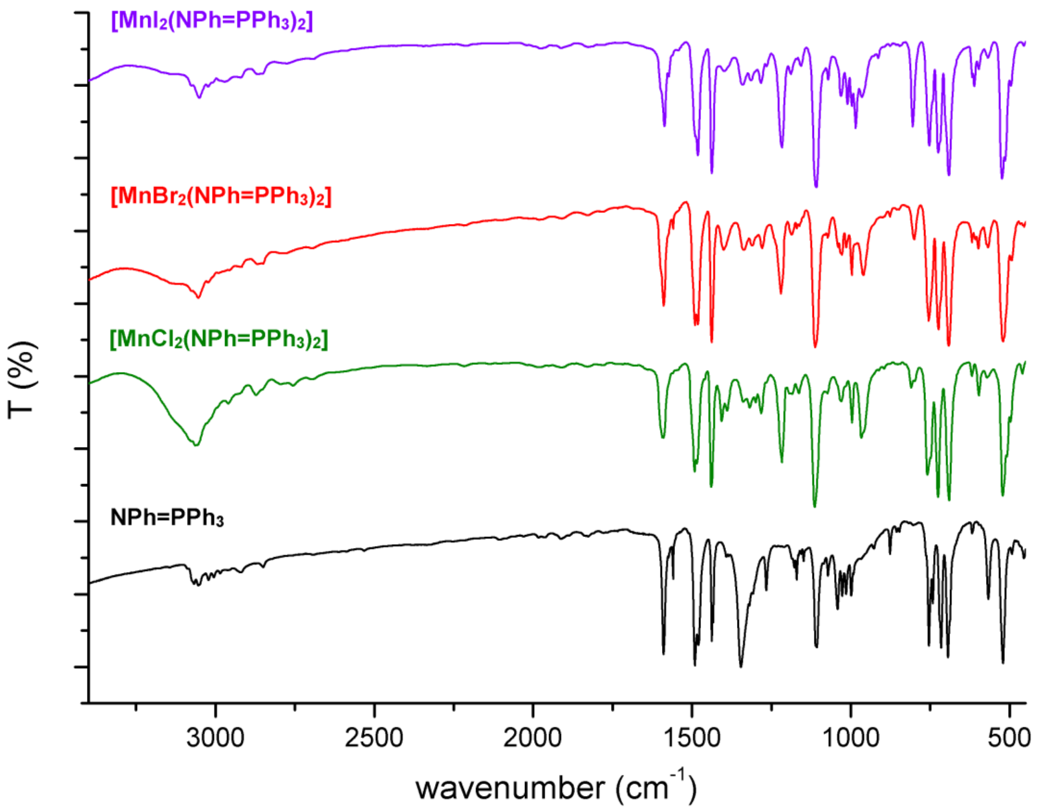

Figure S5. IR spectra (KBr) of $[MnX_2(NPh{=}PPh_3)_2]$ (X = Cl, Br, I) and of $NPh{=}PPh_3$.

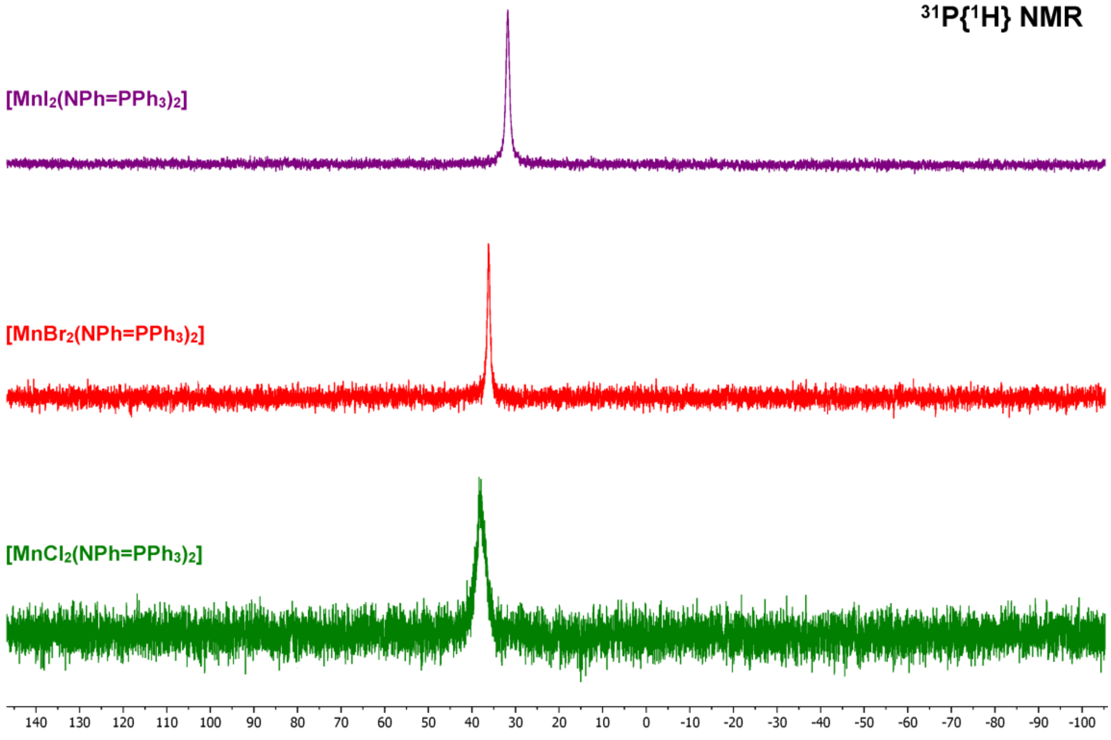

Figure S6. $^{31}P\{^1H\}$ NMR spectra of $[MnX_2(NPh{=}PPh_3)_2]$ (X = Cl, Br, I; $CDCl_3$, 300 K).

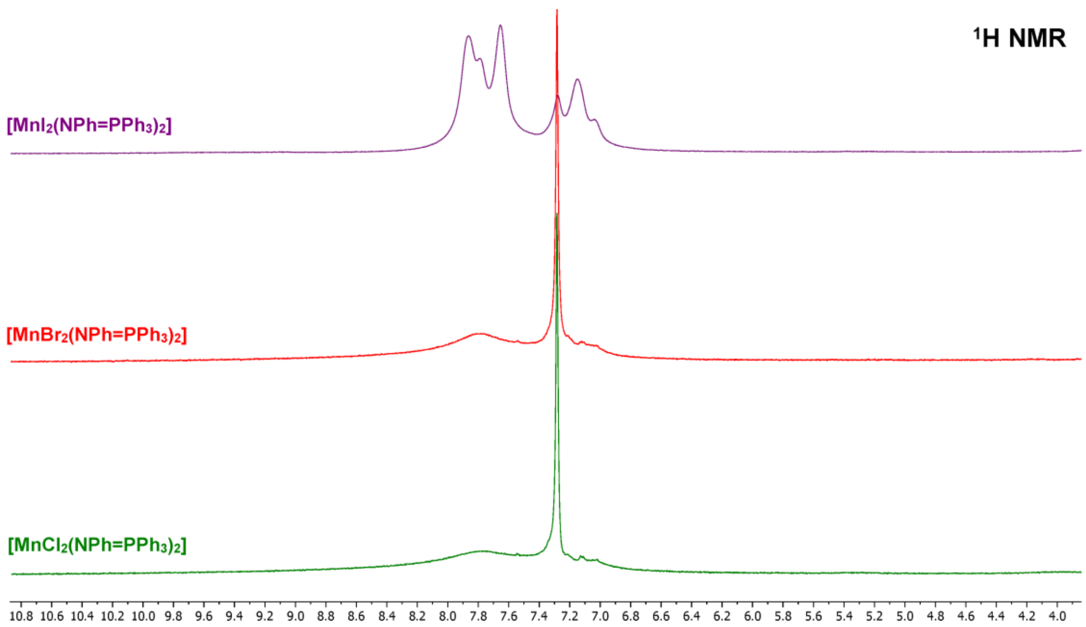

Figure S7. ^1H NMR spectra of $[MnX_2(NPh{=}PPh_3)_2]$ (X = Cl, Br, I; $CDCl_3$, 300 K).

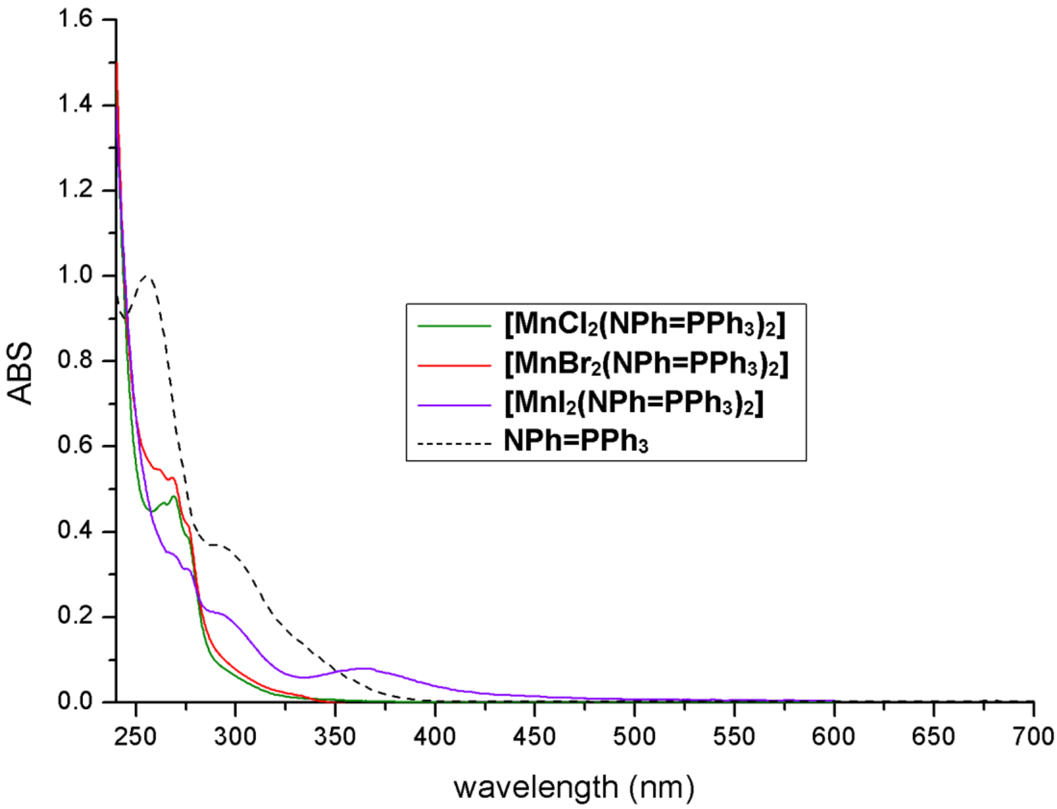

Figure S8. UV-VIS spectra of $[MnX_2(NPh{=}PPh_3)_2]$ (X = Cl, Br, I) and of $NPh{=}PPh_3$ (CH_2Cl_2, r.t.).

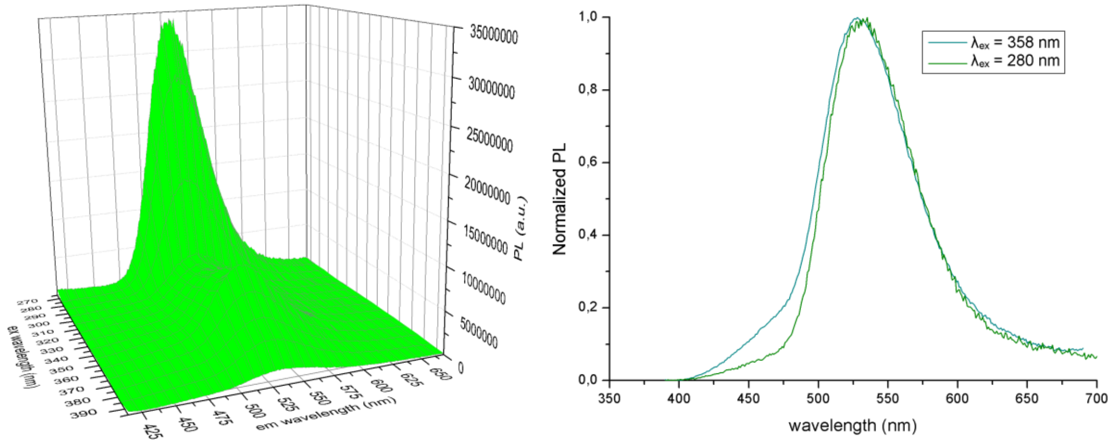

Figure S9. PL spectra of [$MnCl_2(NPh{=}PPh_3)_2$] recorded at different excitation wavelengths (solid, r.t.).

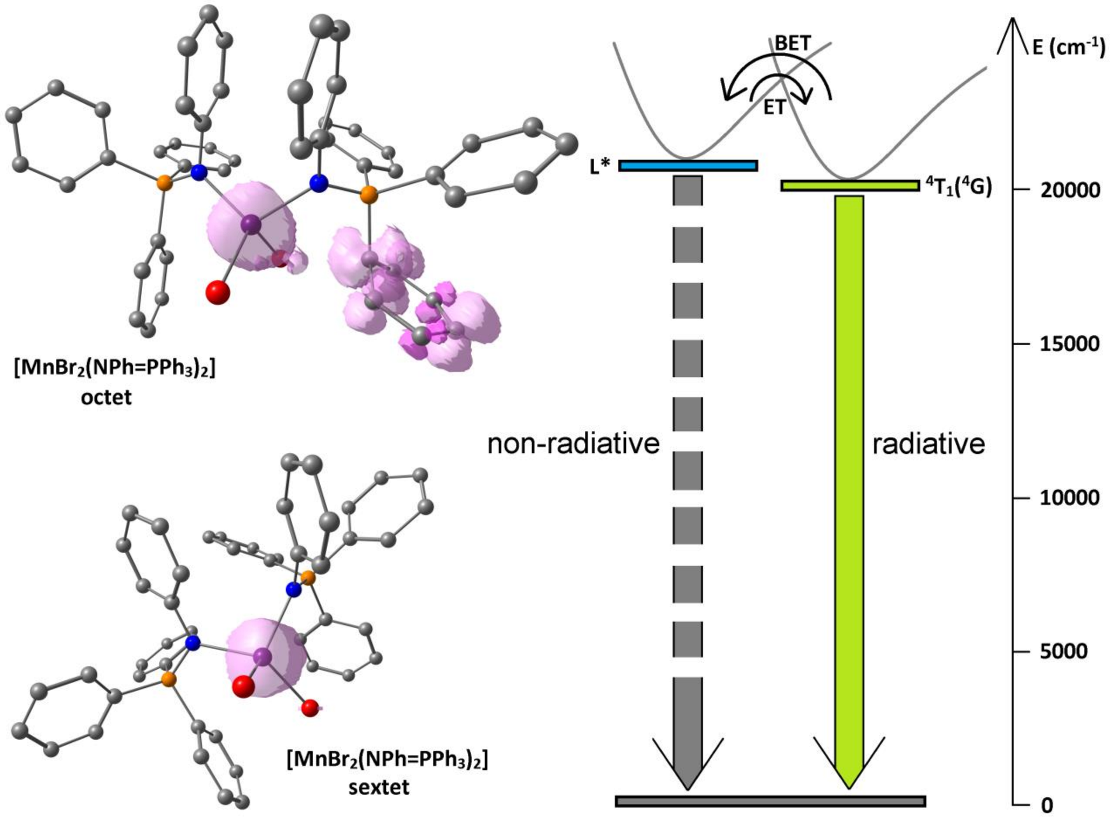

Figure S10. DFT-optimized structures of [$MnBr_2(NPh{=}PPh_3)_2$], sextet and octet configurations, with spin density surfaces (pink tones). Energy difference between the two structures compared to the $^4T_1(^4G)$ — $^6A_1(^6S)$ experimental energy gap. Colour map: Mn, violet; Br, red; P, orange; N, blue; C, grey. Hydrogen atoms are omitted for clarity.

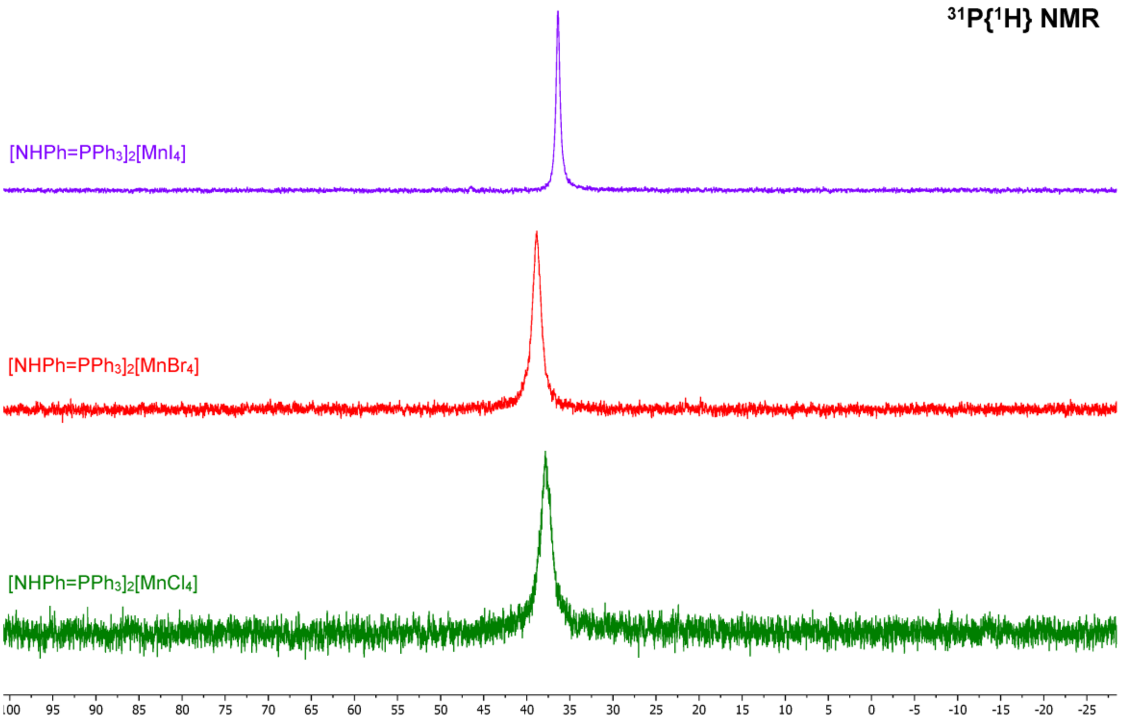

Figure S11. $^{31}P\{^{1}H\}$ NMR spectra of $[NHPh{=}PPh_3]_2[MnX_4]$ (X = Cl, Br, I; $CDCl_3$, 300 K).

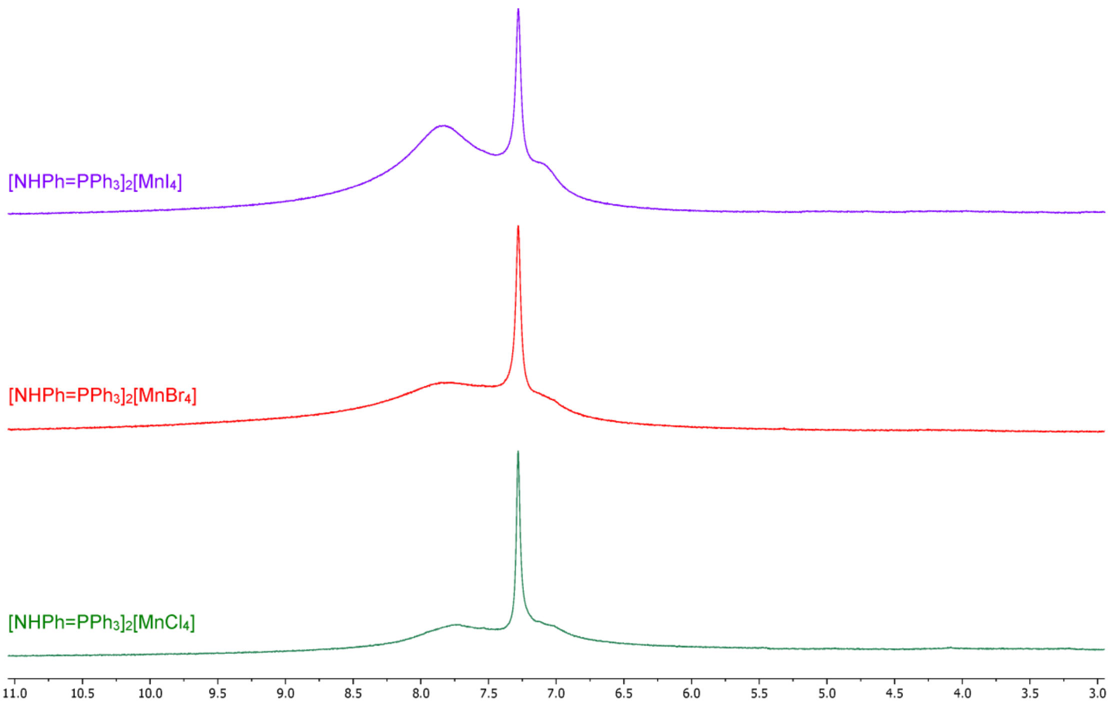

Figure S12. ^{1}H NMR spectra of $[NHPh{=}PPh_3]_2[MnX_4]$ (X = Cl, Br, I; $CDCl_3$, 300 K).

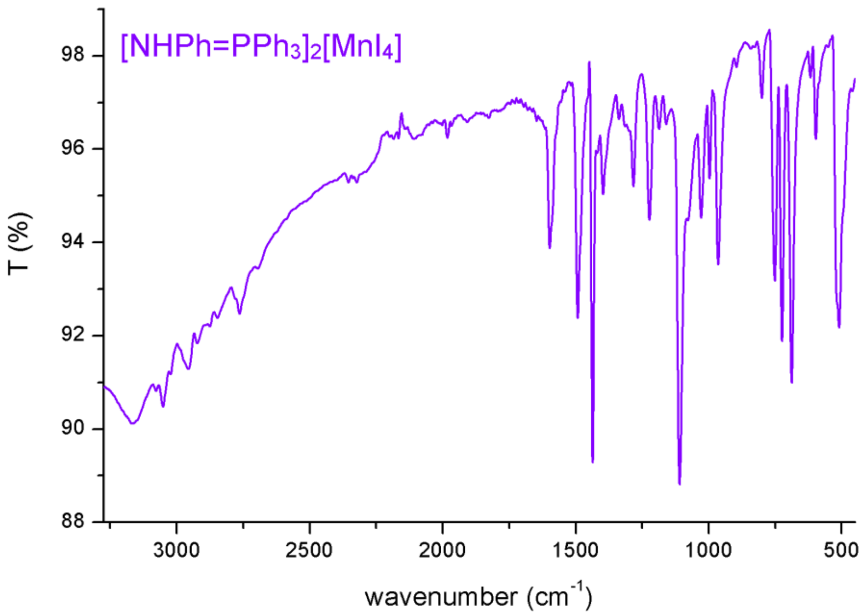

Figure S13. ATR-IR spectrum of [NHPh=PPh$_3$]$_2$[MnI$_4$].

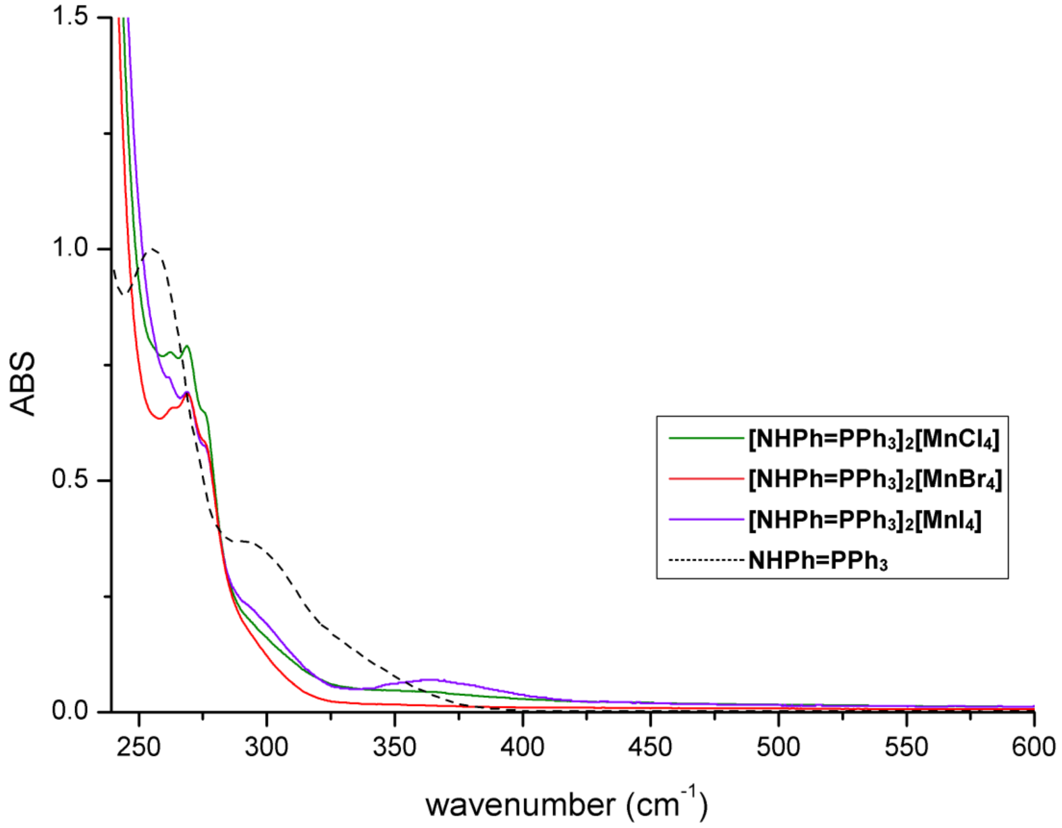

Figure S14. UV-VIS spectra of [NHPh=PPh$_3$]$_2$[MnX$_4$] (X = Cl, Br, I) and of NPh=PPh$_3$ (CH_2Cl_2, r.t.).

Table S1. Output of the SHAPE software and four-coordinate geometry indexes τ_4 and τ'_4.

(*)	SP-4	T-4	SS-4	vTBPY-4	τ_4	τ'_4
Mn	32.123	0.058	8.551	3.117	0.966	0.955
P(1)	32.710	0.131	9.475	3.148	0.972	0.970
P(2)	32.024	0.194	9.239	3.092	0.966	0.964

(*) Studied polyhedrons: SP-4, Square planar; T-4, Tetrahedron; SS-4, Seesaw;vTBPY-4 Vacant trigonal bipyramid. τ_4 and τ'_4 are the descriptor for four-coordinate geometry indexes, extreme forms: 0.00 for SP-4 and 1.00 for T-4.

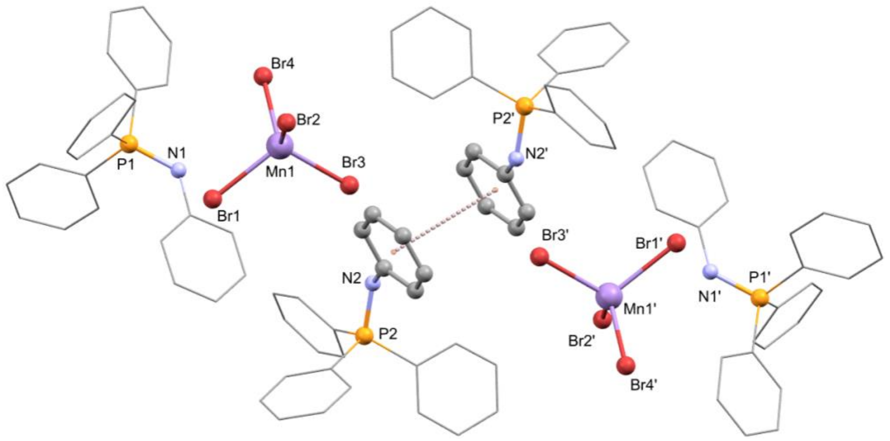

Figure S15. π,π'-stacking interaction. Symmetry operation: i, 1-x, 1-y, 1-z.

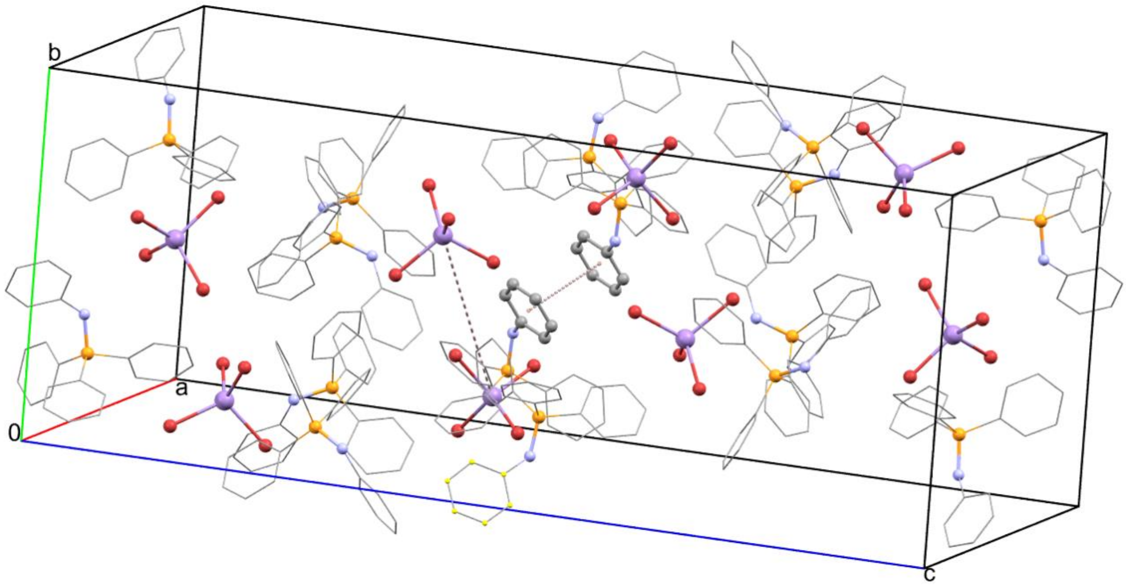

Figure S16. Unit cell content showing the shortest distance between two Mn atoms, 10.573(1) Å.

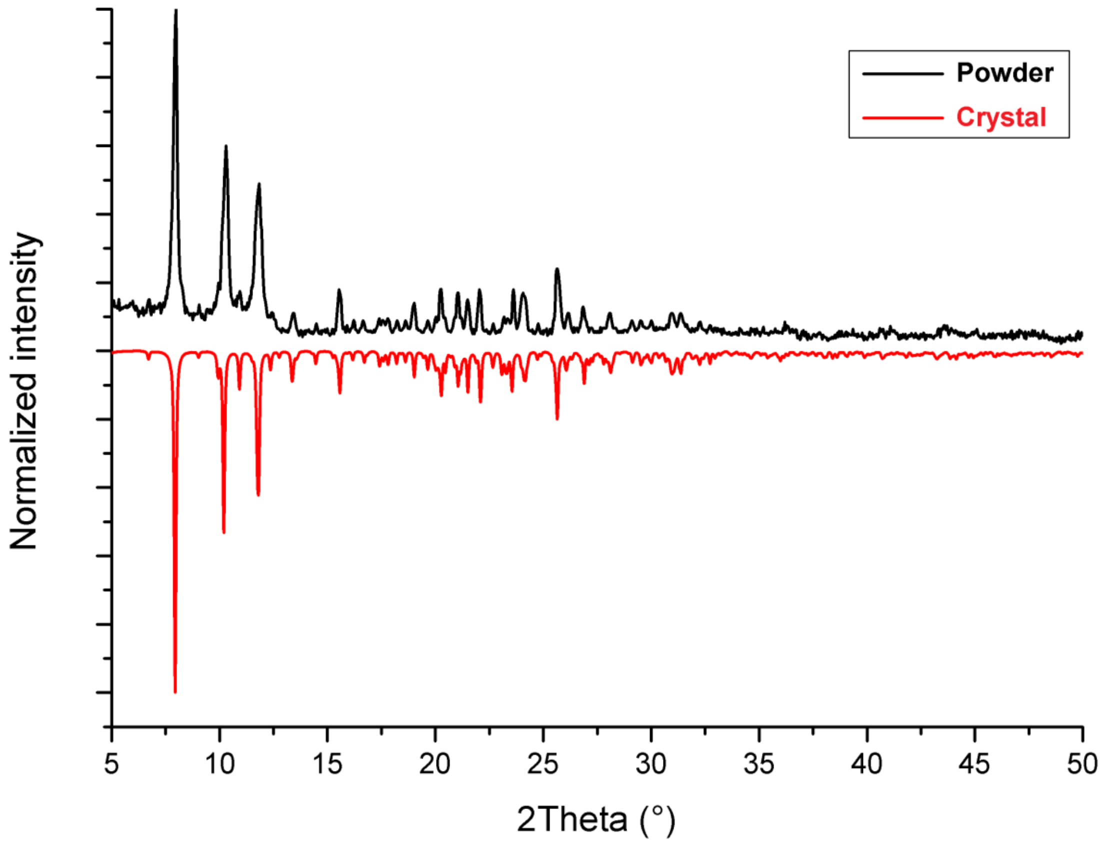

Figure S17. Comparison of the X-ray patterns of $[NHPh{=}PPh_3]_2[MnBr_4]$ obtained from powder (black line) and single crystal (red line) diffraction measurements.

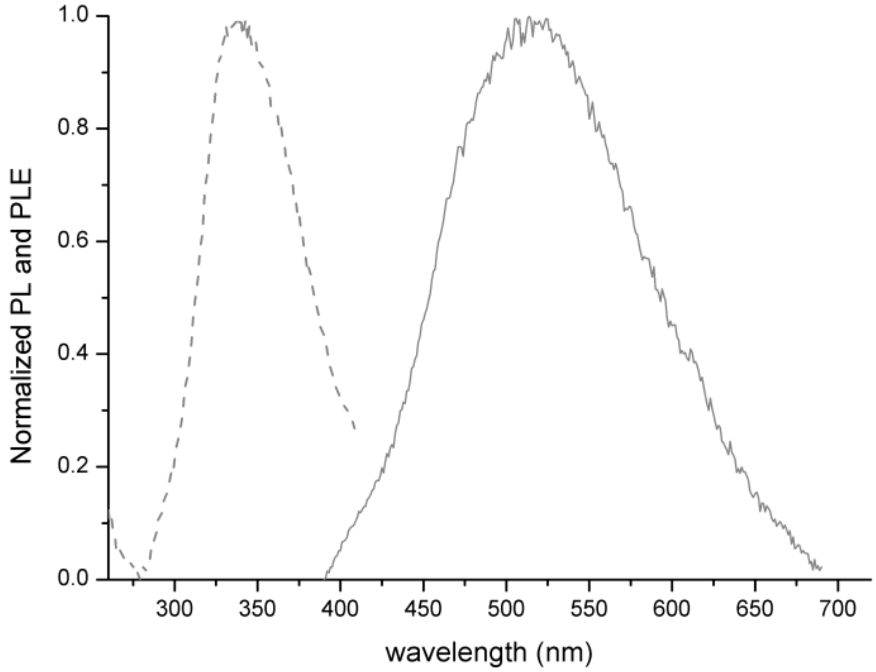

Figure S18. Normalized PL (solid line, λ_{ex} = 350 nm) and PLE (dashed line, λ_{em} = 530 nm) spectra of $[NHPh{=}PPh_3][BF_4]$ (solid, r.t.).

List S1: Cartesian coordinates of NPh=PPh3, ground state (Å).

P	-0.330419000	0.299713000	-0.006769000
C	-1.234660000	-1.263054000	0.228091000
C	-2.567673000	-3.688318000	0.508597000
C	-1.169201000	-2.218747000	-0.781874000
C	-1.990667000	-1.521252000	1.369583000
C	-2.652600000	-2.730470000	1.509531000
C	-1.830008000	-3.429651000	-0.636598000
H	-0.606720000	-2.017215000	-1.684459000
H	-2.079033000	-0.775730000	2.150100000
H	-3.240822000	-2.922711000	2.397074000
H	-1.771355000	-4.168860000	-1.424346000
H	-3.084653000	-4.632606000	0.618286000
C	1.114342000	0.190467000	1.095415000
C	3.423296000	0.124820000	2.641641000
C	1.357509000	-0.885454000	1.942291000
C	2.041476000	1.229006000	1.016303000
C	3.188751000	1.197105000	1.790347000
C	2.509851000	-0.914834000	2.715826000
H	0.657127000	-1.708758000	1.997213000
H	1.866675000	2.054449000	0.337046000
H	3.905826000	2.004210000	1.722265000
H	2.695521000	-1.754597000	3.372310000
H	4.323346000	0.098376000	3.241793000
C	-1.382000000	1.587459000	0.697308000
C	-3.081665000	3.529246000	1.721438000
C	-1.379434000	1.868145000	2.060246000
C	-2.225854000	2.293239000	-0.154919000
C	-3.074773000	3.259633000	0.359659000
C	-2.231616000	2.836560000	2.570327000
H	-0.701978000	1.347068000	2.726381000
H	-2.197188000	2.087705000	-1.216739000
H	-3.729502000	3.808006000	-0.304611000
H	-2.224352000	3.055822000	3.629777000
H	-3.744852000	4.285803000	2.119914000
N	-0.049019000	0.698752000	-1.497438000
C	0.879728000	0.215308000	-2.386908000
C	2.718476000	-0.656366000	-4.349271000
C	0.888515000	0.762789000	-3.679918000
C	1.827594000	-0.783972000	-2.110908000
C	2.728210000	-1.205636000	-3.076857000
C	1.787041000	0.333544000	-4.638542000
H	0.166134000	1.535066000	-3.910230000
H	1.872189000	-1.236113000	-1.126705000
H	3.446348000	-1.976495000	-2.825685000
H	1.760688000	0.778796000	-5.625511000
H	3.421402000	-0.990251000	-5.100474000

List S2: Cartesian coordinates of NPh=PPh3, first singlet excited state (Å).

P	-0.327409000	0.174806000	0.225959000
C	-1.025283000	-1.264361000	0.422000000
C	-2.276656000	-3.517329000	0.583426000
C	-1.858342000	-1.667230000	-0.518574000
C	-0.971192000	-2.009571000	1.493950000
C	-1.521801000	-3.119657000	1.542015000
C	-2.408992000	-2.780502000	-0.451241000
H	-2.013567000	-1.086675000	-1.280211000
H	-0.454113000	-1.694498000	2.250392000
H	-1.407932000	-3.666710000	2.331017000
H	-2.973849000	-3.067733000	-1.181602000
H	-2.733140000	-4.365293000	0.641023000

C	1.121797000	0.326557000	1.126086000
C	3.447872000	0.703996000	2.052129000
C	1.866254000	-0.657772000	1.396112000
C	1.580966000	1.505069000	1.256660000
C	2.709277000	1.695344000	1.783097000
C	2.988121000	-0.471162000	1.945582000
H	1.562705000	-1.550649000	1.212336000
H	1.039243000	2.253903000	0.986678000
H	3.025240000	2.594601000	1.918466000
H	3.517200000	-1.229225000	2.213072000
H	4.339429000	0.844947000	2.389124000
C	-1.358453000	1.512539000	0.628793000
C	-3.024185000	3.336555000	1.143797000
C	-2.052072000	1.453692000	1.685519000
C	-1.511946000	2.471427000	-0.175113000
C	-2.340949000	3.393674000	0.084479000
C	-2.835273000	2.398057000	1.974888000
H	-1.964456000	0.691264000	2.266448000
H	-0.975688000	2.491070000	-0.975140000
H	-2.453134000	4.121385000	-0.537146000
H	-3.322421000	2.380102000	2.805728000
H	-3.671928000	4.023476000	1.337035000
N	0.114719000	0.502794000	-1.169097000
C	0.680750000	0.033399000	-2.065397000
C	2.339048000	-1.059647000	-3.693955000
C	1.470180000	0.765550000	-2.856488000
C	0.660721000	-1.286289000	-2.323212000
C	1.591224000	-1.804517000	-2.973775000
C	2.376996000	0.208516000	-3.503737000
H	1.414658000	1.718624000	-2.763559000
H	-0.008788000	-1.818009000	-1.905632000
H	1.668223000	-2.759612000	-3.033742000
H	3.047636000	0.732482000	-3.949882000
H	2.929309000	-1.457388000	-4.338307000

List S3: Cartesian coordinates of [$MnBr_2(NPh{=}PPh_3)_2$], sextet state (Å).

Mn	0.749833000	-0.053946000	1.056542000
Br	-0.030211000	-2.444830000	1.137580000
Br	2.944592000	0.225470000	2.153442000
N	0.354523000	0.276999000	-1.123565000
N	-0.547439000	1.546421000	1.965211000
P	1.540144000	0.054133000	-2.195250000
P	-0.155887000	3.096103000	2.204173000
C	1.179018000	-1.200113000	-3.453706000
C	0.705921000	-3.239463000	-5.285076000
C	2.210394000	-1.669191000	-4.268539000
C	-0.079041000	-1.789866000	-3.533617000
C	-0.310743000	-2.803741000	-4.451139000
C	1.970879000	-2.676030000	-5.187824000
H	3.215823000	-1.279987000	-4.160036000
H	-0.877860000	-1.483458000	-2.875126000
H	-1.289643000	-3.260851000	-4.499647000
H	2.777299000	-3.035780000	-5.812657000
H	0.520798000	-4.033183000	-5.996746000
C	3.012570000	-0.559804000	-1.378647000
C	5.238272000	-1.613751000	-0.119774000
C	2.979413000	-1.864007000	-0.885315000
C	4.162271000	0.211561000	-1.246660000
C	5.275659000	-0.322514000	-0.620827000
C	4.091164000	-2.381783000	-0.247817000
H	2.084126000	-2.466352000	-0.972495000
H	4.192081000	1.226441000	-1.619967000
H	6.166746000	0.279353000	-0.505725000

H	4.051543000	-3.380535000	0.163575000
H	6.103384000	-2.019421000	0.387736000
C	2.017264000	1.612201000	-3.009015000
C	2.709514000	4.093312000	-4.059229000
C	1.798976000	2.775266000	-2.273537000
C	2.569410000	1.701941000	-4.282657000
C	2.916649000	2.940131000	-4.802610000
C	2.145159000	4.011060000	-2.794347000
H	1.326059000	2.711582000	-1.302115000
H	2.715316000	0.815565000	-4.884248000
H	3.342583000	3.003136000	-5.795035000
H	1.957058000	4.906151000	-2.215902000
H	2.977725000	5.056961000	-4.471964000
C	1.593851000	3.344422000	2.570240000
C	4.296520000	3.684545000	3.120940000
C	2.501380000	3.560423000	1.537532000
C	2.053433000	3.295294000	3.882394000
C	3.399765000	3.463000000	4.154317000
C	3.845617000	3.734761000	1.812625000
H	2.167783000	3.593974000	0.510056000
H	1.369154000	3.129845000	4.702723000
H	3.748974000	3.414834000	5.176667000
H	4.542083000	3.890947000	1.000162000
H	5.349175000	3.810338000	3.336508000
C	-1.094485000	3.773145000	3.601018000
C	-2.527001000	4.702204000	5.796632000
C	-1.228280000	2.979247000	4.740838000
C	-1.695568000	5.026037000	3.565533000
C	-2.406455000	5.489053000	4.662930000
C	-1.938200000	3.445718000	5.833303000
H	-0.796432000	1.986857000	4.768192000
H	-1.632963000	5.643874000	2.680198000
H	-2.874473000	6.463620000	4.624789000
H	-2.042794000	2.819967000	6.709177000
H	-3.088214000	5.062343000	6.648765000
C	-0.442275000	4.206206000	0.791439000
C	-0.891283000	5.892997000	-1.375309000
C	0.092687000	5.498072000	0.780680000
C	-1.191285000	3.767111000	-0.293191000
C	-1.413278000	4.610875000	-1.374345000
C	-0.136992000	6.337651000	-0.295280000
H	0.696356000	5.847803000	1.609332000
H	-1.596595000	2.764431000	-0.310758000
H	-1.991632000	4.250932000	-2.213509000
H	0.276321000	7.337727000	-0.293816000
H	-1.065278000	6.548471000	-2.218728000
C	-1.845515000	1.118936000	2.333531000
C	-4.379677000	0.166110000	3.095661000
C	-3.008599000	1.828202000	2.022548000
C	-1.982706000	-0.073576000	3.046954000
C	-3.232725000	-0.548330000	3.408971000
C	-4.255545000	1.361946000	2.406480000
H	-2.955606000	2.757404000	1.469187000
H	-1.103212000	-0.640998000	3.320723000
H	-3.303017000	-1.485343000	3.945463000
H	-5.135976000	1.939606000	2.155187000
H	-5.354481000	-0.203455000	3.384781000
C	-0.953203000	0.568975000	-1.557143000
C	-3.614343000	1.157619000	-2.302670000
C	-2.040131000	0.108213000	-0.804185000
C	-1.243677000	1.302092000	-2.717898000
C	-2.551577000	1.581338000	-3.084677000
C	-3.342284000	0.414932000	-1.162522000
H	-1.876395000	-0.527334000	0.054850000
H	-0.450257000	1.672691000	-3.351769000

H	-2.732601000	2.142268000	-3.993472000
H	-4.149007000	0.053031000	-0.538810000
H	-4.633632000	1.386554000	-2.583417000

List S4: Cartesian coordinates of [$MnBr_2(NPh{=}PPh_3)_2$], octet state (Å).

Mn	1.252143000	0.677175000	0.827594000
Br	1.629066000	-1.302251000	2.290761000
Br	3.158033000	2.333798000	0.567923000
N	0.576434000	0.210200000	-1.160917000
N	-0.247242000	1.874700000	1.774315000
P	1.503990000	-0.139383000	-2.432916000
P	0.158013000	3.346754000	2.308914000
C	0.870483000	-1.511605000	-3.438778000
C	-0.139606000	-3.624840000	-4.948138000
C	1.636748000	-2.655583000	-3.651345000
C	-0.406743000	-1.431904000	-3.999141000
C	-0.906356000	-2.486619000	-4.744646000
C	1.132062000	-3.705950000	-4.403766000
H	2.631199000	-2.736232000	-3.233723000
H	-1.018935000	-0.551149000	-3.861243000
H	-1.899617000	-2.415890000	-5.166922000
H	1.737098000	-4.588667000	-4.561574000
H	-0.533268000	-4.446642000	-5.531777000
C	3.122943000	-0.609570000	-1.824529000
C	5.575623000	-1.369517000	-0.788392000
C	3.189390000	-1.603539000	-0.849470000
C	4.287123000	-0.002763000	-2.286242000
C	5.511784000	-0.389464000	-1.768263000
C	4.416444000	-1.974647000	-0.327231000
H	2.289632000	-2.078518000	-0.481488000
H	4.241962000	0.778440000	-3.032432000
H	6.416505000	0.089212000	-2.117187000
H	4.454997000	-2.719255000	0.455374000
H	6.533651000	-1.654198000	-0.373922000
C	1.744963000	1.237273000	-3.586171000
C	2.212429000	3.396577000	-5.267356000
C	1.909210000	2.515124000	-3.052432000
C	1.816087000	1.045521000	-4.962881000
C	2.048197000	2.126602000	-5.800728000
C	2.144021000	3.589951000	-3.894090000
H	1.872046000	2.669525000	-1.980858000
H	1.690968000	0.056657000	-5.384348000
H	2.101305000	1.975121000	-6.870574000
H	2.278604000	4.576537000	-3.470773000
H	2.396653000	4.237268000	-5.923368000
C	1.826049000	3.262392000	2.963749000
C	2.682261000	4.101889000	5.458499000
C	2.626807000	4.496437000	3.080127000
C	2.027656000	2.263388000	4.034021000
C	2.346574000	2.729488000	5.264438000
C	2.936679000	4.927249000	4.327742000
H	2.947448000	5.021274000	2.191003000
H	1.885207000	1.207091000	3.842641000
H	2.431333000	2.043686000	6.098668000
H	3.471249000	5.859410000	4.466585000
H	2.911812000	4.462881000	6.451079000
C	-0.985619000	3.868104000	3.609813000
C	-2.652167000	4.558561000	5.728858000
C	-1.183559000	2.979769000	4.669512000
C	-1.646442000	5.092360000	3.612477000
C	-2.473532000	5.435971000	4.670821000
C	-2.011584000	3.327127000	5.721930000

H	-0.705192000	2.009898000	4.663541000
H	-1.539752000	5.775482000	2.781332000
H	-2.986564000	6.388462000	4.661913000
H	-2.162974000	2.629745000	6.534829000
H	-3.300651000	4.827293000	6.552371000
C	0.148835000	4.616306000	1.014960000
C	0.142410000	6.465132000	-1.063629000
C	0.491468000	5.945893000	1.257094000
C	-0.193323000	4.222017000	-0.273181000
C	-0.199511000	5.144220000	-1.308619000
C	0.487144000	6.865164000	0.220235000
H	0.766081000	6.267777000	2.253915000
H	-0.460627000	3.191066000	-0.466734000
H	-0.469523000	4.825643000	-2.307335000
H	0.753833000	7.895343000	0.415316000
H	0.138496000	7.185732000	-1.871067000
C	-1.580879000	1.424301000	1.878686000
C	-4.207776000	0.474648000	2.144843000
C	-2.660865000	2.152263000	1.378573000
C	-1.841571000	0.205475000	2.508562000
C	-3.139251000	-0.261981000	2.635067000
C	-3.957566000	1.683601000	1.515522000
H	-2.489820000	3.091149000	0.867313000
H	-1.016152000	-0.371532000	2.904633000
H	-3.313495000	-1.208224000	3.131794000
H	-4.776558000	2.269477000	1.118335000
H	-5.221527000	0.111518000	2.251823000
C	-0.822018000	0.307136000	-1.351634000
C	-3.594283000	0.429132000	-1.713911000
C	-1.657332000	-0.645423000	-0.771093000
C	-1.399543000	1.334924000	-2.099493000
C	-2.773738000	1.390725000	-2.281588000
C	-3.028869000	-0.582433000	-0.951079000
H	-1.216446000	-1.436750000	-0.177749000
H	-0.765678000	2.083182000	-2.559831000
H	-3.201517000	2.192698000	-2.869844000
H	-3.660260000	-1.326826000	-0.485462000
H	-4.666424000	0.475229000	-1.850363000
